# The sperm quality in DIO male mice is linked to the NF-κB signaling and *Ppp2ca* expression in the hypothalamus

**DOI:** 10.1016/j.isci.2025.112110

**Published:** 2025-02-25

**Authors:** Xu Feng, Maoxing Xu, Ying Liu, Xiaoyu Wang, Yiman Duan, Xiaoyan Zheng, Wen Yin, Yafei Cai, Wei Zhang, Qin Jiang, Jing Pang, Juxue Li

**Affiliations:** 1 College of Animal Science and Technology, Nanjing Agricultural University, Nanjing, Jiangsu 210095, China; 2 State Key Laboratory of Reproductive Medicine and Offspring Health, Nanjing Medical University, Nanjing, Jiangsu 211166, China; 3 Jiangsu Provincial Key Laboratory of Molecular Targets and Intervention of Metabolic Disease, Nanjing Medical University, Nanjing, Jiangsu 211166, China; 4 The Affiliated Eye Hospital, Nanjing Medical University, Nanjing, Jiangsu 210029, China; 5 The Second Affiliated Hospital of Nanjing Medical University, Nanjing, Jiangsu 210011, China; 6 Clinical Center of Reproductive Medicine, Xuzhou Central Hospital, Xuzhou Clinical School of Xuzhou Medical University, Xuzhou, Jiangsu 221000, China

**Keywords:** Molecular biology, Neuroscience

## Abstract

Recent studies show obesity correlated with reduced sperm quality in males, but the mechanism is unclear. In this study, diet-induced obese (DIO) male mice exhibited disrupted luteinizing hormone (LH) pulse release due to altered function of the hypothalamic-pituitary-gonadal (HPG) axis. This alteration was caused by activation of nuclear factor kappa B (NF-κB) signaling in the hypothalamus, which led to decreased sperm quality. RNA sequencing (RNA-seq) analysis of the hypothalamic arcuate nucleus (ARC) revealed a signaling network involving protein phosphatase 2 catalytic subunit alpha (*Ppp2ca*). This network disrupted LH pulse secretion by inhibiting Akt kinase (AKT) and cAMP responsive element-binding protein 1 (CREB1) activities, thereby reducing KiSS-1 metastasis-suppressor (*Kiss1*) expression. Furthermore, overexpression of the *Ppp2ca* gene in the ARC led to disrupted LH patterns and reduced sperm quality. These findings offer new insights into the molecular mechanisms underlying sperm quality decline in DIO male mice.

## Highlights

• Diet-induced obesity (DIO) disrupts the LH pulse pattern in male mice
• Activation of NF-kB signaling in hypothalamus disrupts LH pulse, reducing sperm quality
• *Ppp2ca* upregulation by NF-κB signaling in DIO male mice hypothalamus disrupts LH pulse

## Graphical abstract

**Figure:**
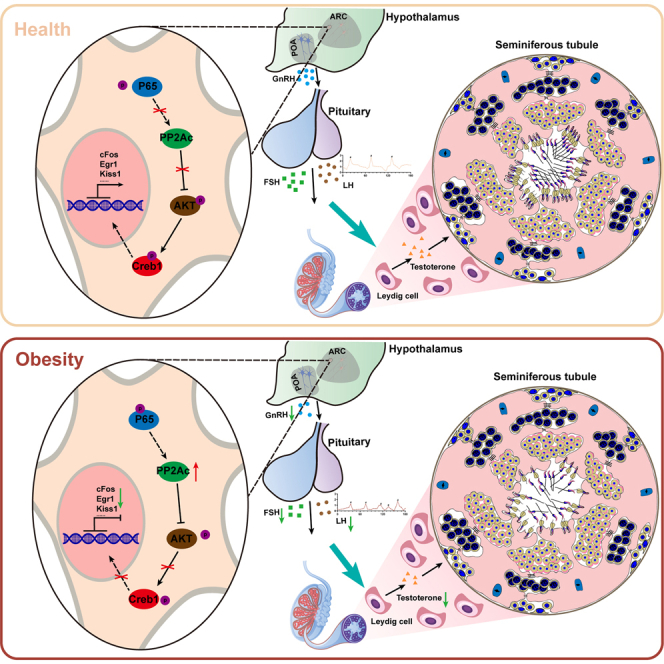

## Introduction

Obesity is a major global public health issue that affects millions of individuals worldwide.^1^ Given its epidemiological prevalence, extensive research has been conducted on the impact of obesity on various biological processes, including reproduction.^2^^,^^3^ These findings indicate a significant correlation between obesity and impaired sperm quality, characterized by decreased sperm count, reduced sperm motility, and abnormal morphology.^4^^,^^5^ The obese male also exhibited abnormal levels of reproductive hormones including luteinizing hormone (LH), testosterone (T), and follicle-stimulating hormone (FSH), which play crucial roles in the regulation of sperm production and maturation.^6^^,^^7^ It is not clear yet to what extent the abnormal reproductive hormones in obese males exert an impact on sperm quality.

The hypothalamic-pituitary-gonadal (HPG) axis plays a vital role in regulating reproduction by orchestrating the secretion of reproductive hormones in both males and females.^8^^,^^9^ In this axis, the hypothalamus acts as a control center for the physiological process of reproductive hormone release.^10^ Kisspeptin released by hypothalamic arcuate kisspeptin/neurokinin B/dynorphin A (KNDy) neurons have been demonstrated to elicit pulsatile gonadotropin-releasing hormone (GnRH) secretion.^11^ GnRH then stimulates the pituitary gland to secrete LH and FSH, which travel through the bloodstream to reach the gonad and regulate the physiological process of germ cell development.^12^^,^^13^ Mounting studies have provided compelling evidences that obesity has been linked to the alternations of LH pulse in females.^14^^,^^15^^,^^16^ An imbalanced release of LH pulses observed among obese women can lead to ovulation disorder such as the polycystic ovary syndrome (PCOS).^17^^,^^18^^,^^19^ Despite the extensive research focus on the impact of obesity on LH pulses in females, there has been limited attention given to males. A clinical study conducted several decades ago demonstrated a significant reduction in the amplitude of LH pulse release among male patients who were obese.^20^ However, the mechanism of dysfunction of HPG, as indicated by abnormal LH pulsatility, to the decline in sperm quality in obese males remains uncertain.

Protein phosphatase 2A catalytic subunit alpha (PP2Ac), encoded by the *Ppp2ca* gene, is a ubiquitously expressed enzyme across mammalian cells, with notably high levels in the brain.^21^ PP2Ac is crucial for neuronal regulation and has been implicated in the modulation of insulin resistance via AKT dephosphorylation.^22^^,^^23^^,^^24^ Studies demonstrate that activation of PP2Ac significantly reduces pAKT levels, disrupting insulin signaling pathways.^25^ Additionally, ceramides have been identified as key mediators that amplify PP2Ac activity, and impair insulin signaling.^26^ These findings primarily derive from research in peripheral tissues, such as muscle and adipose tissue, where PP2Ac contributes to systemic insulin resistance.^26^ Despite its essential role, research exploring the function of PP2Ac within the central nervous system remains limited.

Here, we observed an altered LH pulse release observed in obese male mice is associated with a significant decrease in sperm quality. Moreover, we further revealed that altered LH pulse release in obese mice was link to the activation of nuclear factor kappa B subunit (NF-κB) signaling and *Ppp2ca* expression in the hypothalamus. Our findings provide new insights into the molecular mechanisms that contribute to the decrease in sperm quality observed in male diet-induced obesity (DIO) mice.

## Results

### The DIO induced disruption of LH pulse in male mice

**Figure 1:**
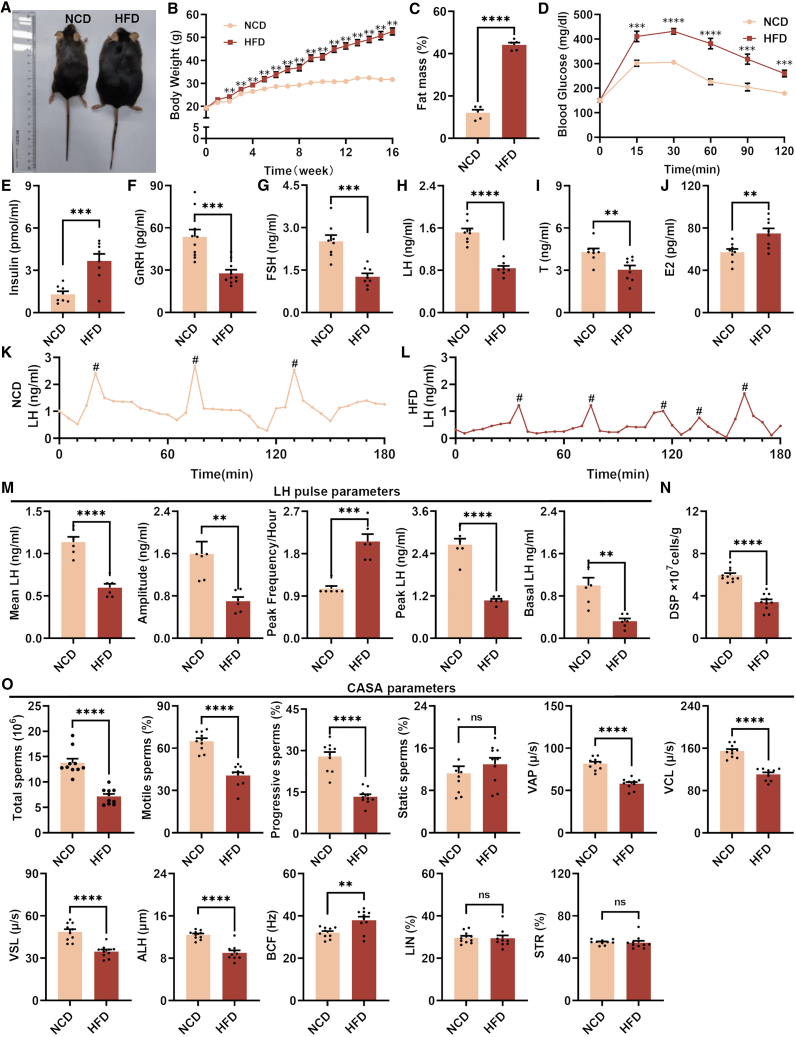
DIO male mice exhibited impaired LH pulse pattern and a decline in sperm quality (A–E) The appearance (A), body weight (B), fat mass (C), glucose tolerance test (D), and serum insulin level (E) of mice feeding on normal-chow diet (NCD) and high-fat diet (HFD) (*n* = 10). (F–J) The levels of gonadotropin-releasing hormone (GnRH) (F), follicle-stimulating hormone (FSH) (G), luteinizing hormone (LH) (H), testosterone (T) (I), and estradiol (E2) (J) in serum of mice feeding on NCD and HFD (*n* = 8). (K and L) The representative LH pulse curves of mice feeding on NCD (K) and HFD (L). (M) LH pulse parameters: mean LH (average LH concentration over a 3-h period for all time points pulse peak), amplitude (average LH concentration of between a pulse peak and its nadir), peak frequency (the number of identified pulse peak), peak LH (average LH concentration of pulse peak), and basal LH (average LH concentration nadir values preceding each pulse peak) in whole blood of mice feeding on NCD and HFD (*n* = 6). # indicates the peak LH concentration. (N) Daily sperm production (DSP) of mice feeding on NCD and HFD (*n* = 10). (O) Total sperms, motile sperms, progressive sperms, VAP, VCL, VSL, ALH, BCF, LIN, and STR of mice feeding on NCD and HFD (*n* = 10). (Total sperms: the number of sperms per mL of medium; motile sperms: percentage of motile sperms; progressive sperms: percentage of forward motile sperm; static sperms: percentage of dead sperm; VAP: mean sperm path rate; VCL: sperm curve rate; VSL: sperm linear rate; ALH: sperm head swing amplitude, BCF: sperm tail whipping frequency; LIN: linearity of sperm movement; STR: forward sperm). Data are presented as mean ± SEM,∗ indicates a significant difference (∗ means *p* < 0.05, ∗∗ means *p* < 0.01, ∗∗∗ means *p* < 0.001, ∗∗∗∗ means *p* < 0.0001), ns indicates non-significant difference, Student’s t test.

In this study, we established a male DIO mouse model by feeding them a high-fat diet (HFD). These mice exhibited typical metabolic characteristics associated with obesity, including significant increases in body weight (Figures 1A and 1B), adipose tissue mass (Figure 1C), blood glucose levels (Figures 1D and S2A), fasting serum leptin level (Figure S2B), and fasting serum insulin level (Figure 1E). We found a significant reduction in serum levels of GnRH, FSH, LH, and T in DIO male mice (Figures 1F–1I), accompanied by a significant increase in estradiol (E2) level and T/E2 ratio (Figures 1J and S2C). Importantly, the DIO male mice exhibited a significant alteration in LH pulse pattern (Figures 1K and 1L), characterized by notable reductions in mean LH amplitude and peak LH amplitude, as well as a decrease in basal LH level (Figure 1M). Additionally, there was a remarkable increase in LH peak frequency (Figure 1M). These findings clearly revealed the dysregulation of reproductive hormone release induced by DIO in male mice. Consistent to previous studies, we have confirmed that the DIO mice exhibited a decreased in sperm quality, including diminished sperm production, reduced sperm motility (Figures 1N and 1O). Within the testis of DIO male mice, the expression of blood-testis barrier related genes, such as intercellular adhesion molecule 1 (*Icam1*), tight junction protein 1 (*Tjp1*) and gap junction protein alpha 1 (*Gja1*) (Figure S2D), and testosterone synthesis related genes, such as hydroxy-delta-5-steroid dehydrogenase 3 beta (*Hsd3β*), hydroxy-delta-5-steroid dehydrogenase 17 beta (*Hsd17β*), steroidogenic acute regulatory protein (*Star*)*,* cytochrome P450 family 11 subfamily A member 1 (*Cyp11a1*), and cytochrome P450 family 11 subfamily B member 1 (*Cyp11b1*) (Figure S2E) were significantly downregulated. However, we observed that the morphology of seminiferous tubules of DIO male mice remains intact, indicating that DIO did not induce any severe damage to seminiferous tubules (Figure S2F).

### NF-κB signaling involved in disruption of LH pulse

**Figure 2:**
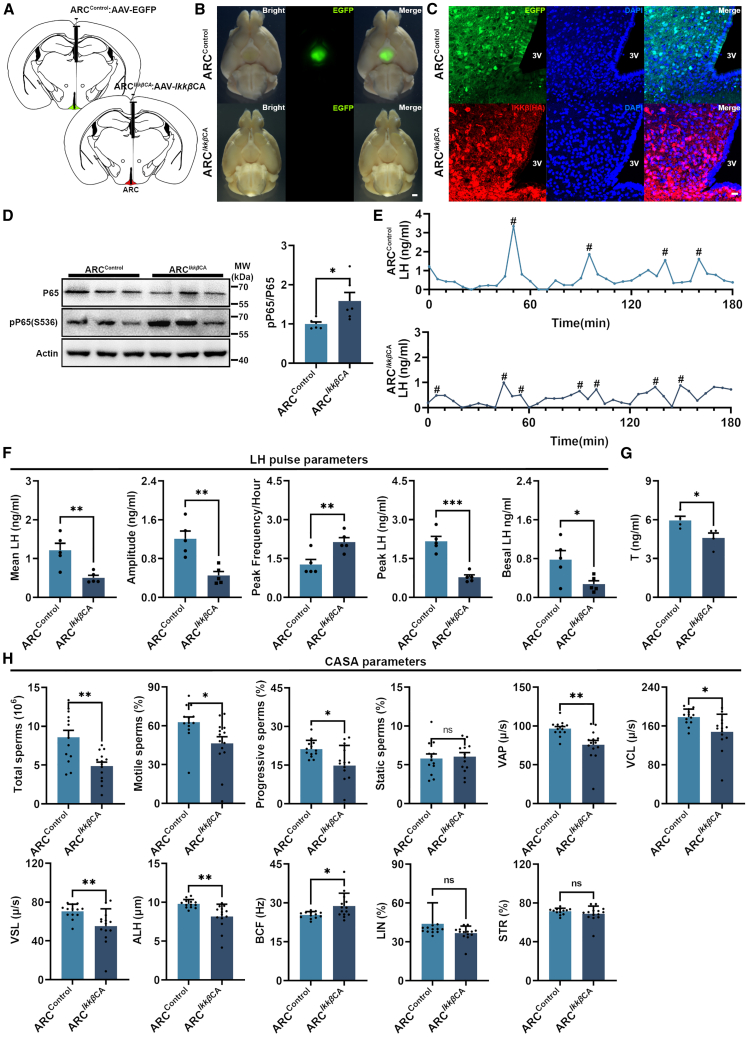
Activation of NF-κB signaling in the hypothalamus resulted in a disrupted LH pulse pattern and a decrease in sperm quality in male mice (A) Schematic depiction of stereotaxic administration of AAV-CAG-EGFP and AAV-CAG-*Ikkβ*CA viral vectors in the mouse hypothalamus. (B) The ventral view of adeno-associated virus (AAV) injected mouse brains under fluorescence microscope, scale bar: 1,000 μm. (C) Representative immunofluorescence images showed the expression of EGFP and *Ikkβ*CA in the arcuate nucleus (ARC) of AAV virus injected mice, scale bar: 20 μm; 3V: Third ventricle. (D) Western blot image and quantification of protein level of P65 and pP65 in hypotahlamus of male mice from ARC^Control^ group and ARC^*Ikkβ*CA^ group (*n* = 3). (E) The representative LH pulse curves of ARC^Control^ and ARC^*Ikkβ*CA^ group male mice, # indicates the peak LH pulse. (F) Mean LH, amplitude, peak frequency, peak LH, and basal LH in whole blood of male mice from ARC^Control^ and ARC^*Ikkβ*CA^ group (*n* = 6). (G) Serum T levels of male mice from ARC^Control^ and ARC^*Ikkβ*CA^ group (*n* = 5). (H) Total sperms, motile sperms, progressive sperms, VAP, VCL, VSL, ALH, BCF, LIN, and STR of male mice from ARC^Control^ (*n* = 13) and ARC^*Ikkβ*CA^ group (*n* = 14). Data are presented as mean ± SEM, ∗ indicates a significant difference (∗ means *p* < 0.05, ∗∗ means *p* < 0.01, ∗∗∗ means *p* < 0.001), “ns” indicates non-significant difference, Student’s t test.

The DIO has previously been demonstrated, in our studies and other investigations, to induce chronic inflammation in the hypothalamus, accompanied by activation of NF-κB signaling.^27^ In our DIO mouse model, we also observed activation of the NF-κB signaling pathway in the hypothalamus (Figure S2G), as evidenced by a significant increase in P65 (RELA proto-oncogene, NF-kB subunit) phosphorylation level. Next, we investigated the potential association between hypothalamic NF-κB signaling pathway and LH pulse release in male mice. To accomplish this, we injected constructively activated i-kappaB kinase beta (IKKβ) (*Ikkβ*CA) adeno-associated virus (AAV) into the arcuate nucleus (ARC) of male mice (referred to as ARC^*Ikkβ*CA^ mice) (Figures 2A–2C).^28^ As anticipated, there was a significant increase in P65 phosphorylation level in the hypothalamus of ARC^*Ikkβ*CA^ male mice (Figure 2D), indicating the activation of NF-κB signaling. The ARC^*Ikkβ*CA^ male mice showed a significant increase of food intake and body weight (Figures S3A and S3B). Similar to the DIO male mice, the ARC^*Ikkβ*CA^ male mice also exhibited a disruption in LH pulse release (Figure 2E), which associated with a significant reduction in mean LH amplitude, peak LH amplitude as well as basal LH level (Figure 2F). Moreover, there was a substantial increase in LH pulse frequency (Figure 2F). The serum T levels in mice also exhibited a significant decrease simultaneously (Figure 2G). Subsequently, we have assessed the sperm quality of ARC^*Ikkβ*CA^ male mice. Our findings indicated that ARC^*Ikkβ*CA^ male mice had impaired sperm quality, characterized by reduced sperm production, decreased sperm motility (Figure 2H). Within the testis of ARC^*Ikkβ*CA^ mice, the expression of blood-testis barrier related genes including *Icam1*, *Tjp1*, and *Gja1* (Figure S3C), and testosterone synthesis related genes including *Hsd3β*, *Hsd17β*, *Star*, *Cyp11a1*, and *Cyp11b1* (Figure S3D) were significantly downregulated. Similar to the DIO mice, the activation of NF-κB signaling did not induce any apparent testicular damage, as evidenced by the preserved integrity of seminiferous tubules observed in ARC^*Ikkβ*CA^ mice (Figure S3E). These findings substantiate our hypothesis that activation of NF-κB signaling in hypothalamus disrupts LH pulse release and leads to diminished sperm quality in male mice.

### RNA-seq screening for genes involved in disruption of LH pulse

**Figure 3:**
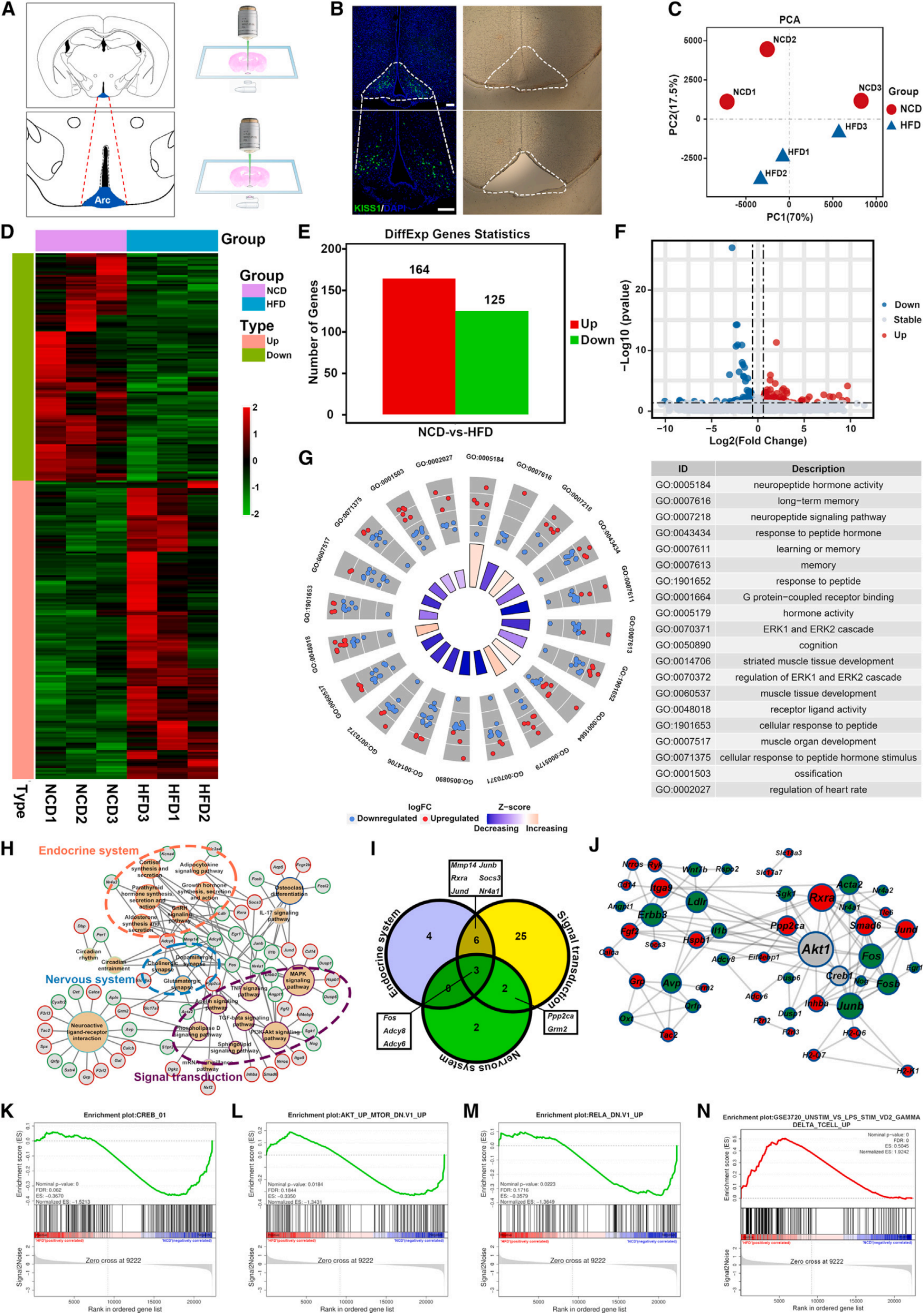
RNA-seq coupled with bioinformatic analysis of ARC enriched with KNDy neurons in DIO male mice (A) Schematic illustration showed laser capture microdissection (LCM) of ARC enriched of Kiss1 neurons. (B) Representative fluorescence image showed the area of ARC enriched of Kiss1 neurons and bright-field image of ARC after LCM, scale bar: 100 μm. (C) Principal-component analysis (PCA) analysis of the RNA-seq data from both NCD group and HFD group. (D) Heatmap of differentially expressed genes (DEGs). (E) Distribution of DEGs in the NCD and HFD groups. (F) Volcano map of DEGs. (G) The top 20 enrichment circles of all gene ontology (GO) terms. (H) The pathway-DEGs network, was constructed based on signal transduction pathway and endocrine system pathway. (I) The distribution of 42 candidate DEGs patway sets including endocrine system, nervous system, and signal tranduction. (J) The protein interaction network of 42 candidate DEGs is represented by circles, with larger circles indicating more protein interactions. Red: upregulated genes, green: downregulated genes, gray: non-significant difference expressed genes. (K–M) The downregulated gene set was regulated by cAMP responsive element binding protein 1 (CREB1) (K), Akt kinase (AKT) (L), and RELA (M) in HFD male mice. (N) The upregulated gene set was regulated by inflammatory activation in HFD male mice.

It is well-established that Kiss1 neurons in the ARC serve as the generator of LH pulses. To investigate the key genes involved in the regulation of LH release, we conducted a RNA sequencing (RNA-seq) study on the ARC region specifically enriched with Kiss1 neurons in DIO male mice. The visualization of Kiss1 neurons in the ARC region was determined by crossing Kiss1-Cre mice with R26R-EYFP reporter mice (Figures 3A and 3B). Laser capture microdissection (LCM) technique was used to precisely isolate the specific regions where Kiss1 neurons are localized in normal-chow diet (NCD) and HFD male mice, followed by RNA extraction for RNA-seq analysis. The gene expression in these samples exhibited distinct clustering patterns as revealed by principal-component analysis (PCA) (Figure 3C), which further illustrated by heatmap and volcano plot (Figures 3D and 3F). Totally, we have identified 389 differentially expressed genes (DEGs), including 164 upregulated genes and 125 downregulated genes (Figures 3E and 3F, Table S1).

To identify candidate genes associated with LH disruption, we conducted enrichment analyses including gene ontology (GO), Kyoto Encyclopedia of Genes and Genomes (KEGG), and gene set enrichment analysis (GSEA). The GO enrichment analysis revealed that the DEGs enriched in various biological functions including metabolic, immune, and reproductive regulation (Figure S4A). Several GO terms related to neuronal cell function were enriched (Table S2), including those associated with interneuronal signaling pathways, such as neuropeptide signaling, hormonal regulation of neurons, and hormone activity (Figure 3G and Table S2). These observed changes in GO terms imply that the neuronal interactions and hormone-regulated functions in the ARC undergo alternations in DIO male mice. The KEGG enrichment analysis revealed that certain DEGs were significantly enriched in the mitogen-activated protein kinase (MAPK) signaling pathway and tumor necrosis factor (TNF) signaling pathway, both of which are closely associated with inflammation and signal transduction^29^^,^^30^ (Figure S4B and Table S3). We constructed an interaction network diagram illustrating the top 50 signaling pathways, which revealed that the DEGs primarily contributed to three categories of signaling pathways: signal transduction, disease, and hormone metabolism (Figure S4C). The signal transduction pathway and endocrine system pathway, which are relevant to the nervous system, were chosen for constructing an interaction network diagram of pathway-DEGs (Figure 3H). Consequently, a final set of 42 candidate genes has been found and used for subsequent analysis (Figure 3I).

Due to the limitations of relying solely on GO and KEGG enrichment analysis for obtaining analytical results from DEGs, it is possible that genes with insignificant expression levels still play crucial biological roles. Thus, GSEA analysis was incorporated as an adjunctive approach to facilitate the identification of potential candidate genes.^31^^,^^32^ The results revealed the upregulation of C2: cgp, C3: tft, and C7 genes in the NCD group, as well as upregulation of C2: cgp, C4, and C7 genes in the HFD group (Table S4). The enrichment analysis revealed a significant inhibition of gene sets regulated by cAMP responsive element binding protein 1 (CREB1) in the HFD group (Figure 3K). Comparatively to other trials (C7), the genes that were upregulated by upregulation of AKT and downregulation of P65 showed a significant decrease in expression levels in the HFD group (Figures 3L and 3M). Additionally, there was an elevation in the expression levels of genes associated with inflammation in the HFD group (Figure 3N). The implications of these findings suggest that the inflammatory responses triggered by DIO lead to a reduction in AKT and CREB1 activity, resulting in differential expression of their downstream related genes. Based on this hypothesis, a protein interaction network was constructed using the Metascape^33^ database and Cytoscape^34^^,^^35^ software, incorporating 42 DEGs as well as AKT and CREB1 (Figure 3J). By integrating the findings of enrichment analysis and the interaction network diagram, we assuming that the expression of *Ppp2ca* gene which encodes the PP2Ac, is upregulated in male HFD mice, leading to inhibition of AKT activity and subsequent decrease in downstream CREB1 activity. This cascade ultimately results in downregulation of FBJ osteosarcoma oncogene (*Fos*), FBJ osteosarcoma oncogene B (*Fosb*), *Kiss1*, and other genes, causing disruption in LH pulse release.

### Upregulation of *Ppp2ca* expression in both DIO and ARC^*Ikkβ*CA^ mice

**Figure 4:**
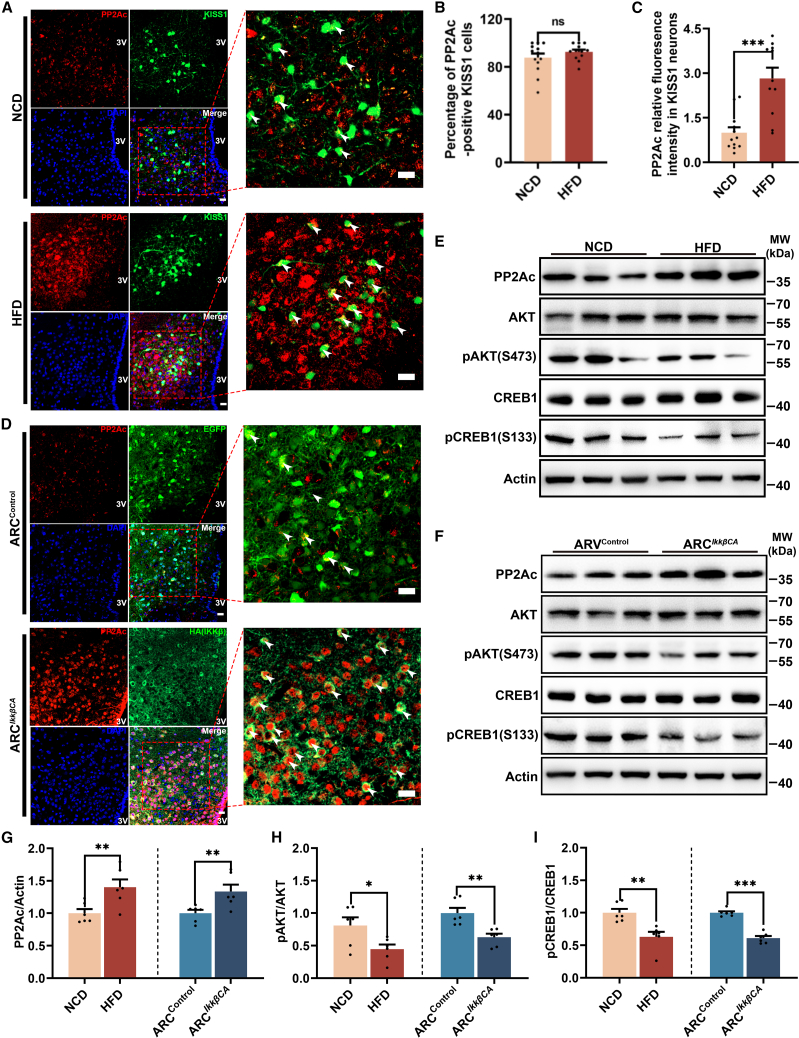
The elevated expression of PP2Ac and decreased activities of AKT and CREB1 in the hypothalamus were confirmed in both DIO mice and ARC^*IKKβ*CA^ mice (A) The representative fluorescence images shows the expression of PP2Ac (Red) in ARC of Kiss1-cre::R26R-EYFP mice under NCD and HFD. The white arrow indicates the expression of PP2Ac in Kiss1 neurons, scale bar: 20 μm. (B) The counting statistics of PP2Ac-positive cells were performed for Kiss1 neurons in the NCD and HFD group (*n* = 3). (C) Relative quantification of PP2Ac in Kiss1 neurons in the NCD and HFD group (*n* = 3). (D) PP2Ac expression in ARC of ARC^Control^ and ARC^*Ikkβ*CA^ group male mice, The white arrow indicates the expression of PP2Ac in AAV-infected neurons, scale bar: 20 μm. (E and F) Western blot analysis of the protein levels of PP2Ac, AKT, pAKT, CREB1, and pCREB1 in the hypothalamus of NCD mice and HFD mice (E) and ARC^Control^ and ARC^*Ikkβ*CA^ mice (F). (G–I) Quantification of protein levels of PP2Ac (G), pAKT (H), and pCREB1 (I) in the hypothalamus of NCD and HFD mice and ARC^Control^ and ARC^*Ikkβ*CA^ mice (*n* = 6). Data are presented as mean ± SEM,∗ indicates a significant difference (∗ means *p* < 0.05, ∗∗ means *p* < 0.01, ∗∗∗ means *p* < 0.001), “ns” indicates non-significant difference, Student’s t test.

To investigate the cellular expression localization of PP2Ac within the ARC, we co-stained PP2Ac with neuronal nuclei (NeuN, a neuronal marker) and ionized calcium-binding adapter molecule 1 (IBA1, a microglia marker) in ARC. Our findings showed that neuronal cells predominantly express PP2Ac, while its presence in microglia is minimal (Figures S4D and S4E). To validate our hypothesis that inflammation-induced upregulation of PP2Ac leads to decreased activity of AKT and CREB1 in the hypothalamus, we first examined PP2Ac expression in both DIO and ARC^*Ikkβ*CA^ mice. The immunofluorescence results revealed an elevated expression of PP2Ac in ARC cells, including Kiss1 neurons, as evidenced by the increased fluorescence signal observed in these cells (Figures 4A–4D). The increased expression of PP2Ac in the hypothalamus was subsequently confirmed by western blot studies in both DIO and ARC^*Ikkβ*CA^ male mice (Figures 4E, 4F, and 4G). Importantly, we observed a significant reduction in the phosphorylation levels of AKT and CREB1 in the hypothalamus of both DIO and ARC^*Ikkβ*CA^ male mice (Figures 4E, 4F, 4H, and 4I). The present findings provide further support for our hypothesis that the involvement of PP2Ac, AKT, and CREB1 is implicated in LH pulse disorder observed in both DIO and ARC^*Ikkβ*CA^ male mice.

### *Ppp2ca* overexpression repression of AKT and CREB1 activities

**Figure 5:**
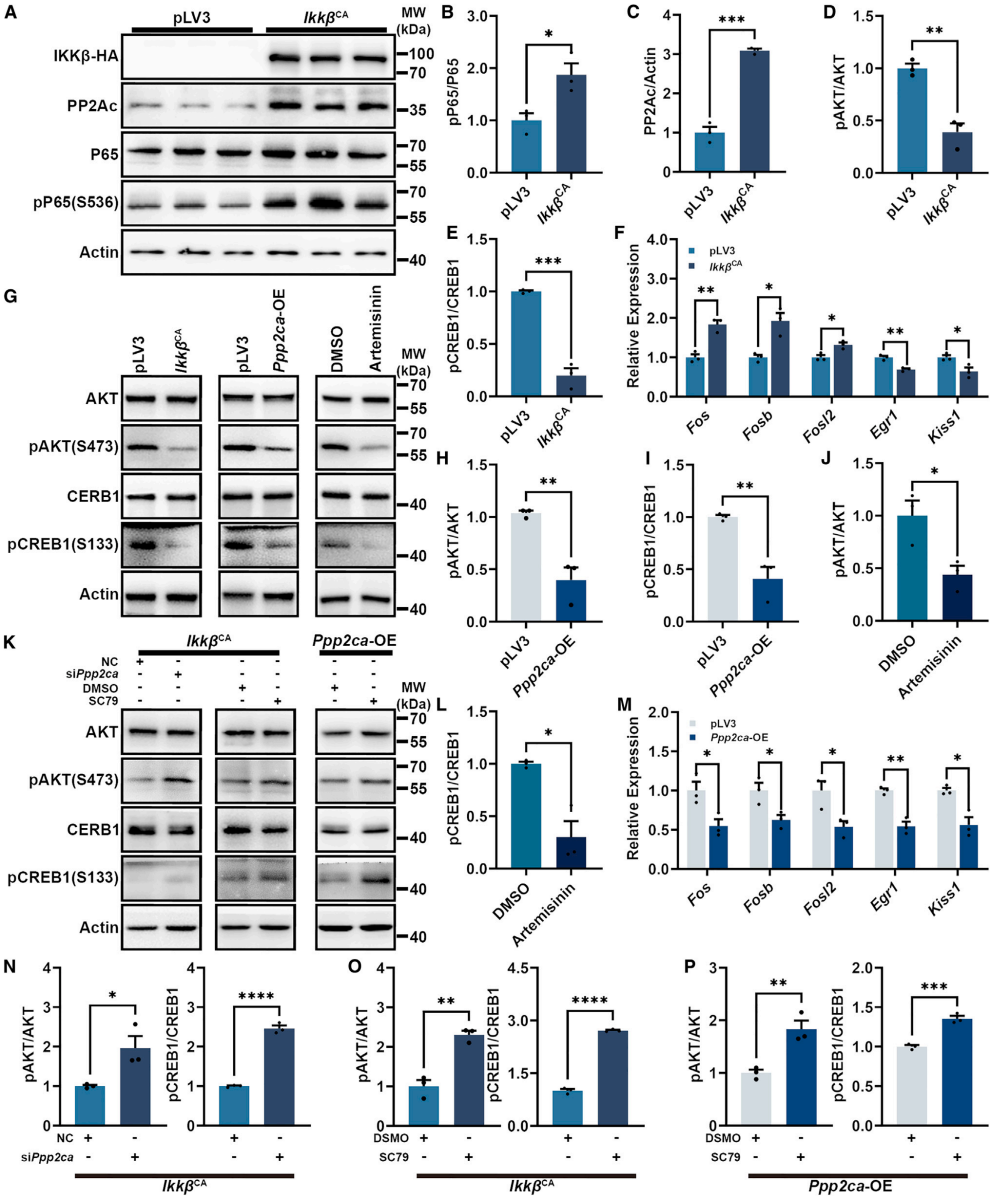
The activation of NF-κB signaling or the overexpression of *Ppp2ca* led to the inhibition of AKT and CREB1 activities (A) Western blot analysis of *Ikkβ***-**HA, PP2Ac, P65, and pP65 in control and *Ikkβ*^CA^ cells. (B–E) The quantification of protein levels of pP65 (B), PP2Ac (C), pAKT (D), and pCREB1 (E) in control and *Ikkβ*^CA^ cells (*n* = 3). (F and M) Relative mRNA levels of *Fos*, *Fosb*, *Fosl2*, *Egr1*, and *Kiss1* in control and *Ikkβ*^CA^ cells (F), control and *Ppp2ca*-OE (M) treated cells (*n* = 3). (G) Western blot analysis of AKT, pAKT, CREB1, and pCREB1 in control and *Ikkβ*^CA^ cells, control and *Ppp2ca*-OE cells and DMSO treated and Artemisinin treated cells. (H–J, and L) The quantification of protein levels of pAKT, pCREB1 in control and *Ppp2ca*-OE cells (H and I) and DMSO treated and Artemisinin treated cells (J and L) (*n* = 3). (K) Western blot analysis of AKT, pAKT, CREB1, and pCREB1 in the *Ikkβ*^CA^ cells treated with NC, si*Ppp2ca*, DMSO, and SC79, as well as in *Ppp2ca-*OE cells treated with DSMO and SC79. (N–P) The quantification of protein levels of pAKT, and pCREB1 in the *Ikkβ*^CA^ cells*,* treated with NC and si*Ppp2ca* (N) (*n* = 3), DMSO and SC79 (O) (*n* = 3), and in the *Ppp2ca*-OE cells treated with DSMO and SC79 (P) (*n* = 3). Data are presented as mean ± SEM,∗ indicates a significant difference (∗ means *p* < 0.05, ∗∗ means *p* < 0.01, ∗∗∗ means *p* < 0.001, ∗∗∗∗ means *p* < 0.0001), Student’s t test.

The MHYPOE-N43/5 cell line (N43/5 cells), a mouse embryonic neuron cell line expressing the *Kiss1* gene, was selected as an *in vitro* model to investigate the key genes involved in the proposed signaling pathway. We established an N43/5 cell line with constitutive activation of NF-κB signaling by expressing a constitutively active form of IKKβ (referred to as the *Ikkβ*^CA^ cell line) (Figure 5A). The activation of NF-κB signaling in *Ikkβ*^CA^ cells was confirmed by the detection of a significantly elevated protein level of pP65 (Figures 5A and 5B). To our expectation, the activation of NF-κB signaling pathway in N43/5 cells led to markedly upregulated PP2Ac protein expression (Figures 5A and 5C). Furthermore, significant reductions in protein levels of pAKT and pCREB1 were observed in *Ikkβ*^CA^ cells (Figures 5G, 5D, and 5E). Interestingly, the mRNA levels of the downstream targeted genes early growth response 1 (*Egr1*), and *Kiss1* were significantly reduced, while the expressions of other targeted genes including *Fos*, *Fosb,* and FOS like 2, AP-1 transcription factor subunit (*Fosl2*) were significantly increased (Figure 5F). The *Fos* gene is an immediate early gene (IEG) that forms heterodimers, specifically the activator protein 1 (AP1), with Jun family proteins to regulate gene expression.^36^^,^^37^ It is expressed in most cell types of the central nervous system and is rapidly induced in response to a variety of acute stimuli, including inflammatory, stress, neuroendocrine, and neuropeptide signals.^38^^,^^39^^,^^40^^,^^41^ However, numerous studies have shown that the level of neuronal *Fos* mRNA is significantly reduced after long-term exposure to chronic stress.^42^^,^^43^^,^^44^ Therefore, we prolonged the culture time of *Ikkβ*^CA^ cells for 5 days and 7 days. The prolong cultured *Ikkβ*^CA^ cells showed a significant reduction in the expression levels of genes, such as *Fos*, *Fosb*, and *Fosl2* (Figures S5C and S5D). To further validate our findings, we established a *Ppp2ca* gene overexpressed N43/5 cell line overexpresses the (*Ppp2ca-*OE cells) (Figure S5A). Consistently, the *Ppp2ca*-OE cells showed a decrease level of pAKT and pCREB1 protein (Figures 5G–5I). Moreover, the mRNA levels of downstream targeted genes including *Fos*, *Fosb*, *Fosl2*, *Egr1*, and *Kiss1* also decreased (Figure 5M).

### The AKT module CREB1 activity in N43/5 cells

The involvement of PP2Ac in the inhibition of AKT and CREB1 phosphorylation has been demonstrated in our study. However, it remains elusive whether the AKT modulate CREB1 activity in our proposed signaling pathway. To address this question, we treated N43/5 cells with the AKT inhibitor Artemisinin^45^ and observed a significant decrease in protein levels of pAKT and pCREB1 (Figures 5G, 5J, and 5L), accompanied by a notable reduction in mRNA levels of *Fos*, *Fosb*, *Fosl2*, *Egr1*, and *Kiss1* (Figure S5E). Interestingly, when the *Ppp2ca* gene was silenced in *Ikkβ*^CA^ cells through treatment with the small interfering RNA of *Ppp2ca* (si*Ppp2ca*) (Figure S5B and Table S7), a significant increase in protein levels of pAKT and pCREB1 was observed (Figures 5K and 5N). Additionally, there was a notable elevation in mRNA levels of *Fos*, *Fosl2*, *Fosb*, *Egr1*, and *Kiss1* (Figure S5F). On the contrary, we also treated both *Ikkβ*^CA^ cells and *Ppp2ca*-OE cells with AKT activator SC79. The results showed a significant increase of protein levels of pAKT and pCREB1 following AKT activation were observed in both cell lines (Figures 5K, 5O, and 5P). Accordingly, mRNA levels of *Fos*, *Fosl2*, *Fosb*, *Egr1*, and *Kiss1* exhibited a significant increase (Figures S5G and S5H). Together, these findings demonstrated that AKT played an important role in module CREB1 activity, subsequently resulting in regulation of the expression of *Fos*, *Fosl2*, *Fosb*, *Egr1*, and *Kiss1.*

### Overexpression of ARC *Ppp2ca* led to disruption of LH pulse

**Figure 6:**
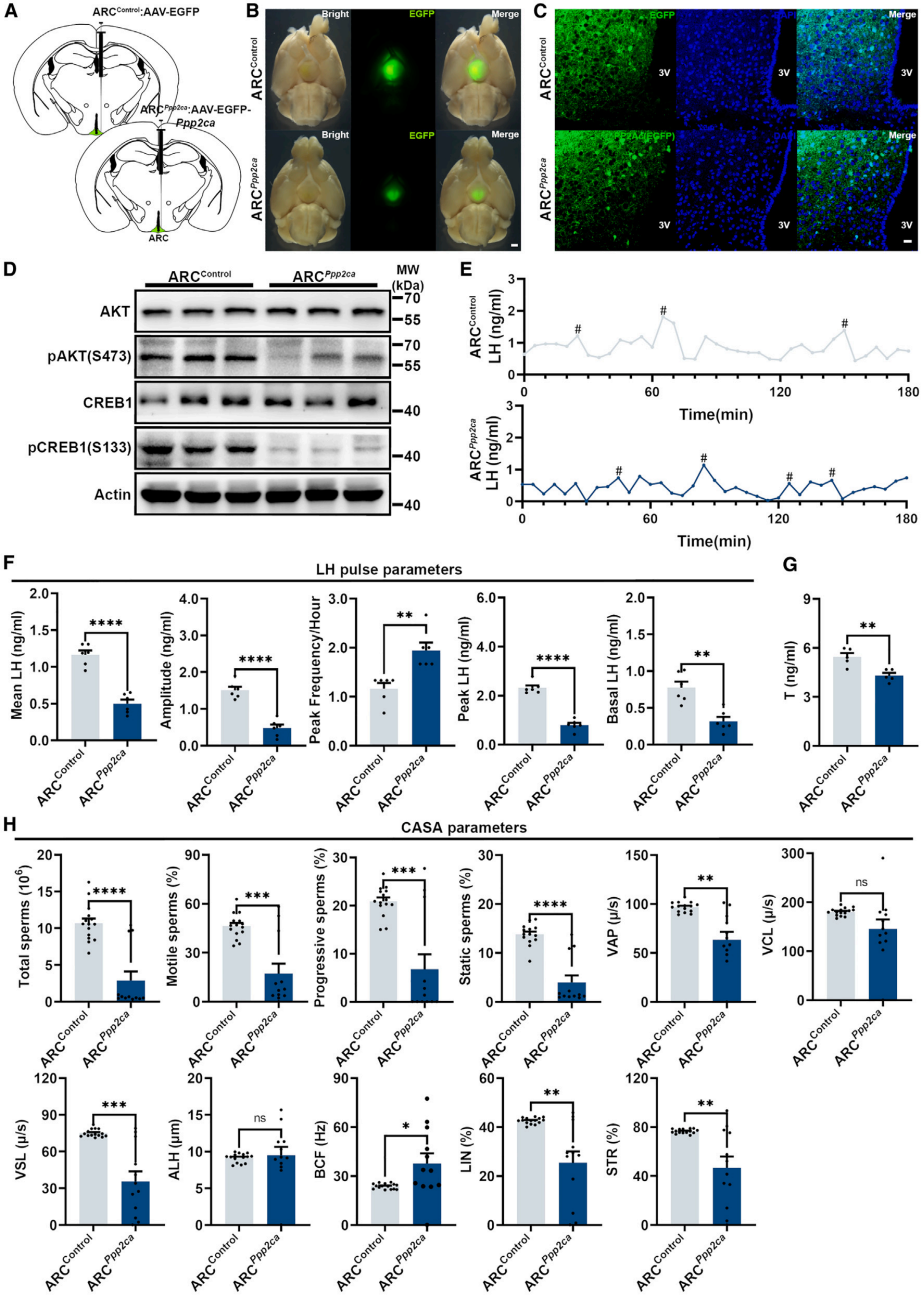
Excessive expression of *Ppp2ca* in the ARC resulted in disturbances to LH pulse patterns and a decline in sperm quality (A) Schematic depiction of stereotaxic injection of AAV-CAG-EGFP and AAV-CAG-EGFP-*Ppp2ca* viral vectors into the mouse hypothalamus. (B) The ventral view of AAV virus injected mouse brains under fluorescence microscope, scale bar:1,000 μm. (C) Representative immunofluorescence images showed the expression of EGFP and PP2Ac in the ARC of AAV virus injected mice, scale bar: 20 μm; 3V: Third ventricle. (D) Western blot analysis of AKT, pAKT, CREB1, and pCREB1 in the hypothalamus of ARC^Control^ and ARC^*Ppp2ca*^ mice. (E) The representative LH pulse curves of ARC^Control^ and ARC^*Ppp2ca*^ mice, # indicates the peak LH concentration. (F) Mean LH, amplitude, peak frequency, peak LH, and basal LH in whole blood of male mice from ARC^Control^ and ARC^*Ppp2ca*^ mice (*n* = 6). (G) Serum T levels of male mice from ARC^Control^ and ARC^*Ppp2ca*^ mice (*n* = 6). (H) Total sperms, motile sperms, progressive sperms, VAP, VCL, VSL, ALH, BCF, LIN, and STR of male mice from ARC^Control^ (*n* = 15) and ARC^*Ppp2ca*^ mice (*n* = 13). Data are presented as mean ± SEM, ∗ indicates a significant difference (∗ means *p* < 0.05, ∗∗ means *p* < 0.01, ∗∗∗ means *p* < 0.001, ∗∗∗∗ means *p* < 0.0001), “ns” indicates non-significant difference, Student’s t test.

To investigate the physiological role of hypothalamic PP2Ac on LH pulse pattern and sperm quality, we established an ARC *Ppp2ca* overexpression mouse model (referred to as the ARC^*Ppp2ca*^ mice) by injection of AAV-CAG-EGFP-*Ppp2ca* into the male mice ARC (Figures 6A–6C). Afterward, we observed a significant reduction in the phosphorylation levels of AKT and CREB1 in the hypothalamus of ARC^*Ppp2ca*^ male mice (Figures 6D and S6A). The ARC^*Ppp2ca*^ mice exhibited significant alterations in peripheral blood LH release pattern (Figure 6E). The mean LH, LH amplitude, and basal LH in the ARC^*Ppp2ca*^ mice were significant decreased (Figure 6F), whereas the peak frequency of LH pulses displayed a notable increase (Figure 6F). The levels of basal LH and peak LH exhibited a significant declining trend, characterized by a reduction in their values (Figure 6F). The peripheral blood testosterone level was significant decrease in ARC^*Ppp2ca*^ mice (Figure 6G). Furthermore, the ARC^*Ppp2ca*^ male mice also exhibited a decline in sperm quality, characterized by reduced sperm production, diminished sperm motility, and increased sperm mortality (Figure 6H). Moreover, qPCR results showed that a significant decrease of mRNA levels of testicular function related genes including *Hsd3β*, *Hsd17β*, *Star*, *Cyp11a1*, and *Cyp11b1* (Figure S6B) and blood-testis barrier-related genes including *Icam1*, *Tjp1*, and *Gja1* were observed in ARC^*Ppp2ca*^ male mice (Figure S6C). We examined the expression changes of *Fos* and other genes in the hypothalamus of ARC^*Ppp2ca*^ male mice. The results revealed a significant reduction in the expression of these genes in the hypothalamus of ARC^*Ikkβ*CA^ and ARC^*Ppp2ca*^ male mice (Figures S6D and S6E). H&E staining results showed that no significant damages observed in the various aspects of ARC^*Ppp2ca*^ mice testes (Figure S6F). Together, these findings indicate that overexpression of *Ppp2ca* in the ARC leads to a disruption in LH pulse pattern and impairment of sperm quality in male mice, which consistent with our findings observed in DIO and ARC^*Ikkβ*CA^ mice.

## Discussion

Obesity, a condition characterized by the excessive accumulation of fat tissue, has emerged as a major public health issue with widespread implications.^46^^,^^47^ In addition to its well-established links to diabetes and cardiovascular disorders, obesity also exerts a substantial influence on reproductive well-being, notably affecting the quality of sperm in males.^48^^,^^49^^,^^50^^,^^51^ Obesity disturbs the delicate hormonal balance necessary for the development of sperm, a process known as spermatogenesis.^52^^,^^53^ One notable factor of its impact resides in the disruptive consequences it has on the HPG axis, a vital regulatory mechanism that governs reproductive function in both males and females.^54^ However, the molecular mechanism underlying the disruption of HPG axis regulation and subsequent decline in sperm quality caused by obesity remains poorly understood.

The present study effectively established a DIO male mouse model that displays typical features of obesity. Similar to previous studies, we have observed abnormal levels of reproductive hormones in the serum, accompanied by compromised sperm quality in these obese mice.^6^^,^^7^^,^^55^ Importantly, we also discover, at the first time, that LH pulse release is disrupted in male obesity mice, indicating dysfunction of the HPG axis. The previous studies conducted by us and others have demonstrated that DIO triggers hypothalamic inflammation, subsequently leading to the dysfunction of hypothalamic neurons and a decrease in various physiological functions controlled by the hypothalamus.^27^^,^^56^ Thus, the proposition put forth in this study posits that hypothalamic inflammation plays a pivotal role in the disruption of LH pulse release. The validation of this hypothesis was demonstrated in the ARC^*Ikkβ*CA^ male mice, which exhibited a disrupted pattern of LH pulse release. Interestingly, the ARC^*Ikkβ*CA^ male mice also exhibited a significantly diminished sperm quality, thereby demonstrating that hypothalamic inflammation resulted in impaired sperm quality.

Kiss1 neurons in the ARC of hypothalamus have emerged as key regulator in controlling HPG by modulating the secretion of GnRH.^57^^,^^58^ The observed decrease in *Kiss1* expression in the hypothalamus of both DIO mice and ARC^*Ikkβ*CA^ male mice has prompted us to investigate the underlying molecular mechanism. By implementing the LCM technique, we successfully isolated Kiss1 neurons enriched in ARC tissues from DIO mice, followed by RNA-seq and bioinformatic analysis. The bioinformatic results led us to construct a signaling network wherein the *Ppp2ca* gene assumes a pivotal regulatory role. The gene *Ppp2ca* encodes the catalytic subunit of protein phosphatase 2A.^59^ PP2A, a prominent phosphatase that acts on serine/threonine residues, consists of three subunits: a scaffold subunit A, a regulatory subunit B, and a catalytic subunit C,^60^ was reported to exerts negative control over the NF-κB, MAPK, and wingless/integrated (Wnt) signaling pathways.^61^^,^^62^^,^^63^^,^^64^ Several studies showed that the promotion of inflammatory bodies’ activation was facilitated by PP2Ac and increase expression of *Ppp2ca* in T cells significantly contribute to molecular defects in systemic lupus erythematosus.^65^^,^^66^^,^^67^ Moreover, PP2Ac has also been reported to mediate the transduction of NF-κB signaling pathway and contribute to the enhancement of inflammatory response.^68^^,^^69^^,^^70^ In the current study, we demonstrated that activation of NF-κB signaling resulted into increase expression of *Ppp2ca* in both N43/5 cells and mouse hypothalamus. The AKT and CREB1, the downstream protein of PP2Ac, also exhibited reduced activities in both N43/5 cells and the mouse hypothalamus upon activation of NF-κB signaling, which was associated with the downregulation of CREB1-targeted genes. Consistent with these results, high expression of *Ppp2ca* led to decrease activities of AKT and CREB1 and decrease expression of CREB1-targeted genes. Additionally, we demonstrated that AKT signaling modulate the CREB1 activity in both N43/5 cells and hypothalamus. Together, through a series of experiments, we validated our bioinformatic analysis proposed signaling network, which aids in comprehending the molecular mechanism underlying the dysfunction of HPG induced by DIO.

In the present study, we explored the physiological role of increase expression of *Ppp2ca* in hypothalamus by establishing the ARC^*Ppp2ca*^ male mice. Interestingly, the ARC^*Ppp2ca*^ male mice showed abnormal LH pulse release and decrease of sperm quality. In addition, there was a decrease in the activities of AKT and CREB1, leading to downregulation of genes targeted by CREB1. These observations align with the findings in DIO and ARC^*Ppp2ca*^ male mice. These results contribute to our understanding of molecular mechanism of the detrimental effects of DIO on sperm quality.

In summary, our study was the first to report that DIO induces alterations in the function of HPG, as evidenced by abnormal LH pulse release, leading to impaired sperm quality. Through RNA-seq coupled bioinformatic analysis and subsequent experimental validation, we have unraveled a signaling network involving NF-κB signaling and *Ppp2ca* expression that responsible for the detrimental effects of DIO on sperm quality. Our discovery provides new insights into the molecular mechanism that underlies the decline in sperm quality caused by DIO.

### Limitations of the study

Previous studies have shown that inflammation-related changes are more closely associated with microglia cells, particularly the NF-κB signaling pathway.^71^ Thus, one limitation of our study is that we primarily focused on investigating the impact of hypothalamic NF-κB signal activation as a whole on LH pulse and sperm quality, while giving less attention to the role of microglia cells in the hypothalamus. In future studies, we are interested in examining inflammation-related changes in various cell types of the hypothalamus of DIO mice and reveal the underlying regulatory mechanisms.

## Resource availability

### Lead contact

Further information and requests for resources and reagents should be directed to and will be fulfilled by the lead contact, Juxue Li (lijuxue@njmu.edu.cn).

### Materials availability

This study did not generate new unique reagents.

### Data and code availability

• The RNA-seq data reported in this paper have been deposited the NCBI GEO Datasets platform (accession number: GSE284408), and are publicly available as of the date of publication.
• Any additional information required to reanalyze the data reported in this paper is available from the lead contact upon request.
• This paper does not report original code.

## Acknowledgments

This research was supported by multiple funding sources, including the Noncommunicable Chronic Diseases-National Science and Technology Major Project（2024ZD0530203), 10.13039/501100001809National Natural Science Foundation of China (grant no. 81570774, 82070872, 92049118, and 82370854), Jiangsu Province’s Innovation Personal as well as Innovative and Entrepreneurial Team of Jiangsu Province (grant no. JSSCTD2021), 10.13039/501100002858China Postdoctoral Science Foundation (grant no. 2023M741790), Jiangsu Funding Program for Excellent Postdoctoral Talent (grant no. 2023ZB558), and Xuzhou Medical Reserve Talents Project (grant no. XWRCHT20220005).

## Author contributions

J.L., conceptualization, funding acquisition, supervision, writing-original draft, and writing-review and editing; J.P., conceptualization, funding acquisition, investigation, and writing-review and editing; Q.J., supervision and writing-review and editing; W.Z., supervision and writing-review and editing; X.F., conceptualization, data curation, investigation, methodology, software, validation, visualization, writing-original draft, and writing-review and editing; M.X., methodology, investigation, and validation; Y.L., funding acquisition and writing-review and editing; X.W., data curation, investigation, methodology, and validation; Y.D., investigation and validation; X.Z., investigation and validation; W.Y., investigation and validation; Y.C., writing-review and editing.

## Declaration of interests

The authors declare no competing interests.

## STAR★Methods

### Key resources table

**Table:** 

REAGENT or RESOURCE	SOURCE	IDENTIFIER
**Antibodies**		

Anti-bovine LH Antibody	University of California	Cat# 518B7; RRID: AB_2665514
Anti-h LH 5303 SPRN-5 Mouse Monoclonal antibody	Medix Biochemica	Cat# 100588; RRID: AB_2784503
HA-Tag Rabbit Monoclonal Antibody	Cell Signaling Technology	Cat# 3724; RRID: AB_1549585
Phospho-Akt (Ser473) Rabbit Monoclonal Antibody	Cell Signaling Technology	Cat# 4060; RRID: AB_2315049
Akt (pan) Rabbit Monoclonal Antibody	Cell Signaling Technology	Cat# 4691; RRID: AB_915783
PP2A C Subunit Rabbit Antibody	Cell Signaling Technology	Cat# 2038; RRID: AB_2169495
NF-κB p65 Rabbit Monoclonal Antibody	Cell Signaling Technology	Cat# 8242; RRID: AB_10859369
Recombinant Anti-Iba1 Mouse Monoclonal Antibody	Abcam	Cat# ab283319; RRID: AB_2924797
Anti-NeuN Mouse Monoclonal Antibody	Abcam	Cat# ab104224; RRID: AB_10711040
CREB1 Rabbit Polyclonal Antibody	Proteintech	Cat# 12208-1-AP; RRID: AB_2245417
Phospho-CREB1 (Ser133) Rabbit Polyclonal Antibody	Bioworlde	Cat# BS79369
PP2A C Subunit Rabbit Antibody	Bioworlde	Cat# BS4867N
Phospho-NF-κB p65 (Ser536) Rabbit Polyclonal antibody	Bioworlde	Cat# BS66162
DDDDK(Flag)-tag Rabbit Polyclonal Antibody	Bioworlde	Cat# AP0007
β-Actin Mouse Monoclonal Antibody	Beyotime	Cat# AF0003; RRID: AB_2893353
α-Tubulin Rabbit Polyclonal Antibody	Beyotime	Cat# AF0001; RRID: AB_2922414
HRP-labeled Goat Anti-Rabbit lgG (H+L)	Beyotime	Cat# A0208; RRID: AB_2892644
HRP-labeled Goat Anti-Mouse IgG (H+L)	Beyotime	Cat# A0216; RRID: AB_2860575
Goat Anti-Rabbit IgG (H+L) Highly Cross-Adsorbed Secondary Antibody, Alexa Fluor Plus 555	Thermo Fisher Scientific	Cat# A32732; RRID: AB_2633281
Goat anti-Mouse IgG (H+L) Highly Cross-Adsorbed Secondary Antibody, Alexa Fluor Plus 488	Thermo Fisher Scientific	Cat# A32723; RRID: AB_2633275

**Chemicals, peptides, and recombinant proteins**		

Roswell Park Memorial Institute (RPMI) 1640	Gibco	Cat# C11875500BT
Dulbecco’s Modified Eagle Medium (DMED)	Gibco	Cat# C11995500BT
Penicillin Streptomycin	Gibco	Cat# 15140122
Fetal bovine serum (FBS)	ExCell	Cat# FSP500
Artemisinin	MCE	Cat# HY-B0094
SC79	MCE	Cat# HY-18749
Trizol reagent	Takara	Cat# T9108
SYBR Green Master Mix	Vazyme	Cat# Q331-03

**Oligonucleotides**		

qPCR primers used for expression analysis	This Paper	See Table S6
Small interfering RNAs (siRNAs) used for silence the *Ppp2ca*	This Paper	See Table S7

**Bacterial and virus strains**		

pAAV-CAG-GFP	addgene	Cat# 37825
pAAV2/9n	addgene	Cat# 112865
pAdDeltaF6	addgene	Cat# 112867
pMDLg/pRRE	addgene	Cat# 12251
pRSV-Rev	addgene	Cat# 12253
pCMV-VSV-G	addgene	Cat# 8454
pLV3-CMV-MCS-3×HA-Puro	Miaolingbio	Cat# P30780

**Critical commercial assays**		

Mouse Leptin ELISA Kit	Crystal Chem	Cat# 90030
Mouse Insulin ELISA Kit	biogenic	Cat# MS100
Gonadotropin-releasing hormone Assay Kit	JianCheng	Cat# H297
Luteinizing hormone Assay Kit	JianCheng	Cat# H206-1
Follicle stimulating hormone Assay Kit	JianCheng	Cat# H101-1
Testosterone Assay Kit	JianCheng	Cat# H090-1
Estradiol 2 Assay Kit	JianCheng	Cat# H102
Dual-labeled three-color multiple fluorescence staining reagent Kit	AiFang biological	Cat# AFIHC023
Mycoplasma Stain Assay Kit	Beyotime	Cat# C0296

**Experimental models: Cell lines**		

MHYPOE-N43/5 cell line	Fenghbio	Cat# CL0733
HEK-293FT cell line	BNCC	Cat# BNCC342056
AAV-293 cell line	Cobioer	Cat# CBP60863

**Experimental models: Organisms/strains**		

B6.129X1-Gt (ROSA)26Sor^tm1(EYFP)Cos^/J (R26R-EYFP)	The Jackson Laboratory	RRID: IMSR_JAX: 006148
STOCK Kiss1^tm1.1(cre/EGFP) Stei^/J (Kiss1-cre)	The Jackson Laboratory	RRID: IMSR_JAX: 017701
C57BL/6J	Laboratory Animal Center of Nanjing Medical University	N/A

**Software and algorithms**		

ImageJ	Schindelin et al.^72^	Version 1.53c
Graphpad	BioMed-Soft	Version 9.4.1
GSEA	Subramanian et al.^32^ and Mootha et al.^31^	Version 4.3.2
PulsarOtago	Porteous et al.^73^	https://pulsar.otago.ac.nz
SPSS	IBM	Version 26.0
Cytoscape	Shannon et al.^34^ and Otasek et al.^35^	Version 3.8.0

**Deposited data**		

RNA sequencing raw data	This paper	Accession number: GSE284408

### Experimental model and study participant details

#### Animals

R26R-EYFP (JAX stock #006148), Kiss1-Cre (JAX stock #017701) were purchased from the Jackson Laboratory. The C57BL/6J wild type mice utilized in this study were obtained from the Laboratory Animal Center of Nanjing Medical University. All mice were housed under specific pathogen-free (SPF) conditions, with a temperature maintained at 22-25°C and humidity ranging from 40% to 70%. A light-dark cycle of 12 hours each was implemented, and no more than five mice were kept per cage. All animal experiments conducted in this study have received approval by the Animal Care and Use Committee of Nanjing Medical University and adhere to relevant ethical requirements (IACUC: 2001012).

#### Cell lines

N43/5 cells (Fenghbio, catalog No: CL0733) were cultured in Roswell Park Memorial Institute (RPMI) 1640 medium(Gibco, catalog No: C11875500BT) supplemented with 10% FBS (ExCell, catalog No: FSP500) and 1% of 10,000 U/mL Penicillin Streptomycin (P/S) (Gibco, catalog No: 15140122), under conditions of 37°C and 5% CO_2._ HEK-293FT cells (BNCC, catalog NO: BNCC342056) and AAV-293 cells (Cobioer, catalog NO: CBP60863) were cultured in Dulbecco’s Modified Eagle Medium(DMED, Gibco, catalog No:C11995500BT) supplemented with 10% FBS (ExCell, catalog No: FSP500) and 1% of 10,000 U/mL Penicillin Streptomycin (P/S) (Gibco, catalog No: 15140122), under conditions of 37°C and 5% CO_2._ Cell lines were routinely tested for mycoplasma contamination using Mycoplasma Stain Assay Kit(Beyotime, catalog No: C0296)

### Method details

#### DIO mice model and measurement of relevant metabolic parameters

Four weeks old male mice were fed either an ordinary diet (NCD; 16.5% fat, cobio, catalog No:1010086) or a high-fat diet (HFD; 60% fat, Research Diets, catalog No: D12492) for 16 weeks. Mouse body weight was measured every week. For the glucose tolerance test (GTT), mice were intravenously administered D-glucose (1.25 g·kg^−1^, Sigma, catalog NO: G8644) following an overnight fasting period. Blood samples were collected from the tail vein at 0, 15, 30, 60, 90 and 120 minutes post-injection to measure glucose levels. Glucose measurements were conducted using a glucometer (ACCU-CHEK Aviva Plus System, Aviva). The body composition of mice was assessed by employing a body composition analyzer (MGAG-MED, China, AccuFat-1050).

#### Virus production and injection

The coding sequence (CDS) of the constitutively active fragment of IKKβ (*Ikkβ*CA) and *Ppp2ca* was inserted into the pAAV-CAG-EGFP vector (Addgene, catalog No: 37825) (Figure S1A) to generate the pAAV-CAG-*Ikkβ*CA-3×HA and pAAV-CAG-EGFP-*Ppp2ca*-3×HA (Figures S1B and S1C). The CDS of *Ikkβ*CA and *Ppp2ca* was inserted into the pLV3-CMV-MCS*-*3×HA-Puro vector (Miaolingbio, catalog NO: P30780) (Figure S1D) to create the pLV3-CMV-*IkkβCA*-3×HA-Puro and pLV3-CMV-*Ppp2ca*-3×Flag-Puro (Figures S1E and S1F).

For lentivirus production, HEK-293FT cells were co-transfected with the packaged plasmids including pMDLg/pRRE (addgene, catalog No: 12251), pRSV-Rev (addgene, catalog No: 12253), pCMV-VSV-G (addgene, catalog No: 8454), along with the pLV3-CMV-*Ikkβ*CA-3×HA-Puro or pLV3-CMV-*Ppp2ca*-3×FLAG-Puro (Figure S1). The cell culture supernatant containing released lentiviral particles was collected after 72 hours transfection.

The AAV virus production procedure was described previously.^74^ Briefly, AAV-293 cells were transfected with packaging plasmids pAAV2/9n (addgene, catalog No: 112865) and pAdDeltaF6 (addgene, catalog No: 112867), along with the core plasmid pAAV-CAG-GFP or pAAV-CAG-*Ikkβ*CA or pAAV-CAG-GFP-*Ppp2ca*. The cell culture medium was replaced 10 hours post-transfection, and the transfected cells, along with the cell culture supernatant, were collected after an additional 72 hours culure period. Iodixanol density-gradient ultracentrifugation was utilized for purification and concentration of virus. The concentrated AAVs were diluted with PBS to achieve a concentration of 10^13^ vg·mL^−1^ and stored at -80°C. The virus was injected into 8 weeks old male C57BL/6J wild type mice, which were subsequently allowed to freely eat and drink for a period of 8 weeks. After that, the mice were sacrificed and samples were collected.

#### Stereotaxic surgery

For the stereotactic surgery, the male mice were anesthetized with isoflurane and securely positioned onto a stereotaxic frame (RWD Life Science, China). Following a small incision to expose the skull, where two small holes were drilled, an injection needle was carefully inserted into the brain. A total of 0.5 μL of AAV virus was delivered at a rate of 25 nL per minute using a micro syringe pump (RWD Life Science, China). The specific coordinates used for the injection site were as follows: anterior-posterior [AP], -1.50 mm; dorsal-ventral [DV], -5.85 mm; left-right [LR], ±0.30 mm).

#### Measurement of reproductive hormones

Prior to the experiment, mice were subjected to a 12-hour fasting period while having access to water. Subsequently, their eyeballs underwent surgical removal and blood samples were collected. Approximately 300-500 μL of blood was obtained and allowed to agglutinate at 4°C for 3-6 hours. The blood samples were then centrifuged at 825g for 15 minutes, resulting in separation into supernatant and pellet fractions. The supernatant was meticulously transferred into new 1.5 mL EP tubes. Following the instructions provided by each Enzyme-Linked Immunosorbent Assay (ELISA) kit manufacturer (Leptin: Crystal Chem, catalog No: 90030; Insulin: biogenic, catalog No: MS100; GnRH: JianCheng, catalog No: H297; LH: JianCheng, catalog No: H206-1; FSH: JianCheng, catalog No: H101-1; Testosterone: JianCheng, catalog No: H090-1; E2: JianCheng, catalog No: H102), serum levels of Leptin, Insulin, GnRH, LH, FSH, T and E2 were measured.

The LH pulse measurement protocol employed in this study follows the methodology reported previously.^75^^,^^76^ Briefly, blood samples were collected from the tail tip of mouse and mixed with a PBST solution (0.05% Tween20 in PBS), then frozen using dry ice. Blood samples were obtained every 5 minutes for a total duration of 180 minutes. A high-affinity 96-well plate (Croning, catalog No: 9018) was coated with LHβ capture antibody solution (UC Davis, catalog No: 518B7) at a concentration of 1 ng·μL^−1^. Following an incubation period of 16 hours at 4°C without agitation, the plates underwent washing and blocking with skim milk powder solution at room temperature for 3 hours. Subsequently, after another round of washing, the plates received additions of blood samples and standards (National Hormone and Peptide Program, AFP5306A), followed by incubation at room temperature for a duration of 2 hours. Then, further washing occurred before introducing biotinylated LHβ detection antibody (Medix Biochemica, catalog No: 100588) to the plates which were then incubated again at room temperature for 90 minutes. Afterwards, the plates underwent another round of washing before adding diluted PBST solution containing poly-HRP conjugate streptavidin (1:8000) (Thermo Fisher, catalog No: N200), which was then incubated once more at room temperature for an additional 90 minutes. Following this step, the plates experienced another cycle of washing prior to shading them with O-Phenylenediamine Dihydrochloride (OPD) (Thermo, catalog No: 34005) solution containing H_2_O_2_ (0.1%) at a concentration level of 0.5 mg·mL^−1^; this shading process took place under controlled conditions set at 37°C for 30 minutes. Finally, LH concentration analysis was conducted using full-wavelength microplate reader (Molecular devices, America) with absorbance measurements taken utilizing wavelengths set a 490 nm and 655 nm. The evaluation pertaining to LH pulse characteristic indices was carried out employing PulsarOtago software (https://pulsar.otago.ac.nz) following the methodology described by a previous study.^73^

#### Immunofluorescence

The brain tissues were collected and fixed in 4% paraformaldehyde (PFA) solution. They were then embedded in OCT compound and sliced into sections measuring 20 μm in thickness. The sections were subjected to a 1-hour treatment with a blocking agent consisting of 5% bovine serum albumin (BSA), followed by an overnight incubation at 4°C with primary antibodies (Table S5). After rinse with PBS for three times, fluorophore conjugated secondary antibodies were administered to the sections for 2 hours at room temperature. Subsequently, the sections underwent another round of washing and were counterstained with DAPI (Solarbio, catalog No: C0065). The Tyramide Signal Amplification (TSA) technique (AiFang biological, catalog No: AFIHC023) was employed to enhance the staining scheme in cases where co-staining of primary antibodies from the same species was necessary. Images were captured using a laser scanning confocal microscope (ZEISS, Germany).

#### Computer-aided sperm analysis

To evaluate different sperm parameters, we conducted computer-assisted analysis on mice sperm. In brief, the mice were euthanized and their epididymal tails were carefully dissected, finely sectioned, and placed in 0.5 mL of preheated DMEM (Gibco, catalog No: C11995500BT). The samples were then incubated at 37°C for 5 minutes to ensure complete release of sperm. Subsequently, gentle agitation and mixing took place before extracting a volume of 10 μL for analysis using the IVOS II automatic sperm analyzer (Hamilton Thorne Biosciences, America). The assessed parameters encompassed total count of sperm cells, motility of sperm cells, rate of forward progressive motility, as well as percentage of non-viable sperm cells.

To assess the daily production of sperm (DSP), we employed a well-established methodology.^77^^,^^78^ In brief, unilateral extraction of testicular tissue from mice was performed, followed by dissection and weighing of the white membrane. Subsequently, 1.5 mL of homogenate buffer (comprising 0.9% NaCl and 0.05% Triton X-100) was added to the tissue and homogenized using a homogenizer. The resulting homogenized sample was then diluted at a ratio of 1:10 in buffer solution before mixing with 100 μL of sample diluent combined with an equal volume of 0.04% Trypan blue dye for incubation at room temperature for 90 minutes. Under an optical microscope equipped with a hemocytometer, elongated sperm cells were enumerated through duplicate counts conducted on three distinct squares per count repetition. The final daily sperm count can be calculated as (sperm cell count ∗20∗1.5) /4.84.

#### Hematoxylin-eosin (H&E) staining

The testes were immersed in Bouin’s fixation solution overnight, followed by paraffin embedding and sectioning into 5 μm slices using a vibratome machine. The sections underwent dewaxing with xylene and rehydration with gradient ethanol before being stained with hematoxylin and eosin. Subsequently, the images were captured using an optical microscope (Nikon, Japan).

#### Quantitative PCR

The Trizol reagent (Takara, catalog No: T9108) was employed to extract total RNA from tissues and cells, following the protocol provided by the manufacturer. Afterwards, cDNA synthesis was performed on the RNA samples using PrimeScript RT (Vazyme, catalog No: R323-01). The resulting cDNA was then analyzed via qPCR on an ABI QuantStudio7 instrument (Life Technologies, America), utilizing SYBR Green Master Mix (Vazyme, catalog No: Q331-03). Please refer to Table S6 for the primer sequences.

#### Western blotting

The proteins were obtained from hypothalamic tissues or cell cultures, subjected to SDS-PAGE separation, and subsequently transferred onto a PVDF membrane (Millipore, catalog No: IPVH00010). Following this, the membrane was blocked using 5% skim milk and exposed to an antibody targeting (Table S5). After incubation with horseradish peroxidase-conjugated secondary antibodies (Table S5). The specific bands corresponding to the target proteins were visualized using the ECL Western blotting detection system (Tanon, China). ImageJ software (Version 1.53c) was utilized for quantifying relative protein expression levels.

#### LCM of ARC and RNA-seq analysis

The brains of mice were harvested and thoroughly washed in cold PBS solution until complete removal of any residual blood on their surfaces was achieved. Subsequently, they were preserved by embedding them in OCT compound and storing them at -80°C. Brain tissue sections measuring 20 μm thickness were obtained using a Microtome Cryostat (Thermo, America). These sections were securely mounted onto slides coated with laser microcut membrane (Leica, 11505190). A laser microdissection system (Leica, Germany) was employed to precise excision ARC regions within the brain sections. The harvest tissues were collected into a RNase-free tube and storage at -80°C.

The RNA extracted from the ARC sample was fragmented to obtain segmented mRNA, which served as the template for synthesizing the first strand of cDNA. Subsequently, the cDNA was synthesized through a dNTPs reaction by using. The NovaSeq6000 platform (Illumina, America) was used to acquire the raw data. The clean reads were obtained by Fastq.^79^ The short read comparison tool bowtie2 was used to compare clean reads with the mouse ribosome database.^80^ HISAT2 was utilized for comparative analysis based on the mouse reference genome, classifying the genomic regions into exonic, intronic, and intergenic regions in conjunction with species possessing comprehensive data.^81^ Subsequently, StringTie and RSEM were employed to calculate expression levels of HISAT2 results for gene read count data as well as FPKM values for subsequent differential expression analysis.^82^^,^^83^ DESeq2 was employed to analyze expression levels while differentially expressed genes (DEGs) were identified using a threshold of P-value < 0.05 and |FC| > 1.5.^84^

#### Bio-informatics analysis

To perform GO and KEGG enrichment analyses, the clusterProfiler and org.Mm.eg.db packages were utilized to analyze the differentially expressed genes. The GSEA (Version 4.3.2) analysis was incorporated to provide supplementary information for the screening of candidate genes. Enrichment thresholds were set at *P* < 0.05, FDR < 0.25, and |ES| > 1. Gene enrichment profiles were visualized using the stringr, dplyr, ggplot2, and ggrepel packages. The obtained enrichment results were then utilized to identify relevant signaling pathways. Circular graphs for GO enrichment analysis, interaction graphs for KEGG signaling pathways, and network graphs for Pathway-DEGs interactions were generated using the Enrichplot package. Network relationships of candidate genes were retrieved from the Metascape database (https://metascape.org/). Protein interaction network diagram illustrating the selected genes was plotted using Cytoscape (Version 3.8.0).

#### siRNA and reagents

Three siRNAs were designed based on the CDS region of the *Ppp2ca* gene by using DSIR.^85^^,^^86^ (http://biodev.extra.cea.fr/DSIR/DSIR.html), and their sequences are listed in Table S7. Cells were transfected with Lipofectamine 2000 when they reached approximately 70% confluence, following the manufacturer’s instructions. Subsequently, cells were collected at 24 and 48 hours post-transfection for RNA and protein extraction. The addition of SC79 (10.96 μM) (MCE, catalog No: HY-18749) and Artemisinin (50 μM) (MCE, catalog No: HY-B0094) occurred at 70% confluence of the cells, followed by cell harvesting after 24 hours of culture.

### Quantification and statistical analysis

The data were subjected to statistical analysis using SPSS software (version 26.0, SPSS), and the results were presented as Mean ± SEM. The normality test has been successfully applied to all the data. If the data passes Levene’s test for homogeneity of variances, a student’s t-test assuming equal variances is employed; otherwise, a student’s t-test assuming unequal variances is utilized. Statistical significance was indicated by asterisks (∗ means *P* < 0.05, ∗∗ means *P* < 0.01, ∗∗∗ means *P* < 0.001, ∗∗∗∗ means *P* < 0.0001), while "ns" represented no significant differences.

## Supplemental information

Document S1. Figures S1–S6 and Tables S4–S7

Table S1. DEGs between the NCD and HFD group male mice

Table S2. GO enrichment analysis result of DEGs

Table S3. KEGG enrichment result of DEGs
