# Supplementary material for: The sperm quality in DIO male mice is linked to the NF-κB signaling and Ppp2ca expression in the hypothalamus
Source: iScience. 2025 Feb 25;28(3):112110. doi: 10.1016/j.isci.2025.112110 (PMC11951025; doi:10.1016/j.isci.2025.112110)
Supplement: Document S1. Figures S1–S6 and Tables S4–S7 [file mmc1.pdf]

iScience, Volume *28*

Supplemental information

The sperm quality in DIO male mice is linked to the NF-κB signaling and *Ppp2ca* expression in the hypothalamus

Xu Feng, Maoxing Xu, Ying Liu, Xiaoyu Wang, Yiman Duan, Xiaoyan Zheng, Wen Yin, Yafei Cai, Wei Zhang, Qin Jiang, Jing Pang, and Juxue Li

Supporting Information

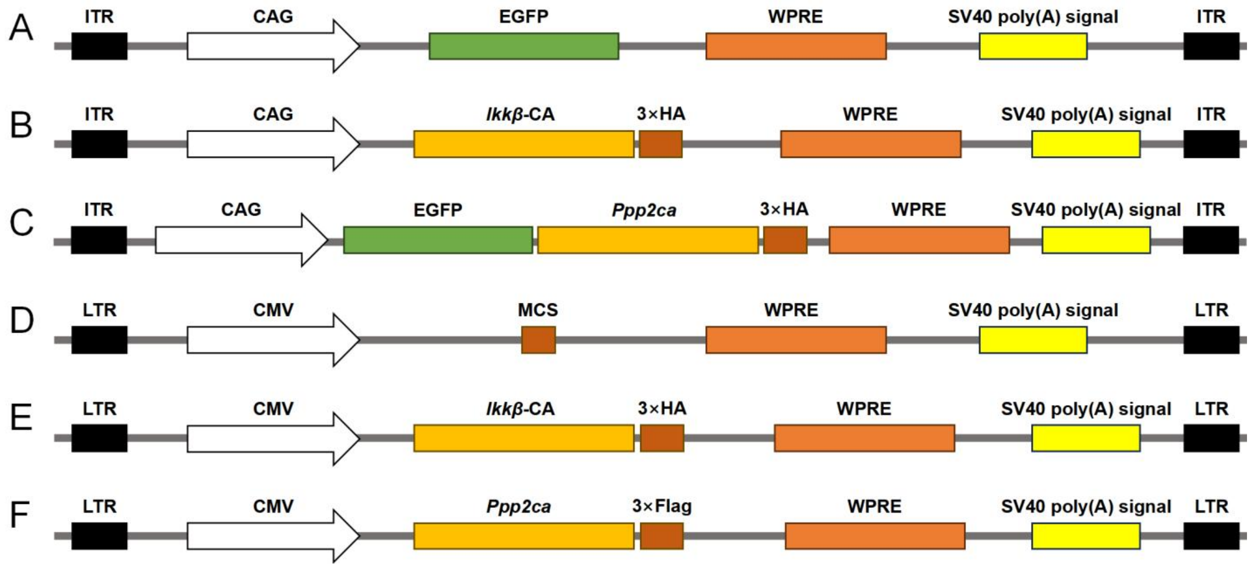

Supplemental Figure 1 Schematic representation of the structure of the virus core plasmid
(A-F) The schematic diagrams of pAAV-CAG-EGFP (A), pAAV-CAG-*Ikkβ*CA-3×HA (B), pAAV-CAG-EGFP-*Ppp2ca*-3×HA (C), pLV3-CMV-Puro (D), pLV3-CMV-IkkβCA-3×HA-Puro (E), pLV3-CMV-Ppp2ca-3×Flag-Puro (F).

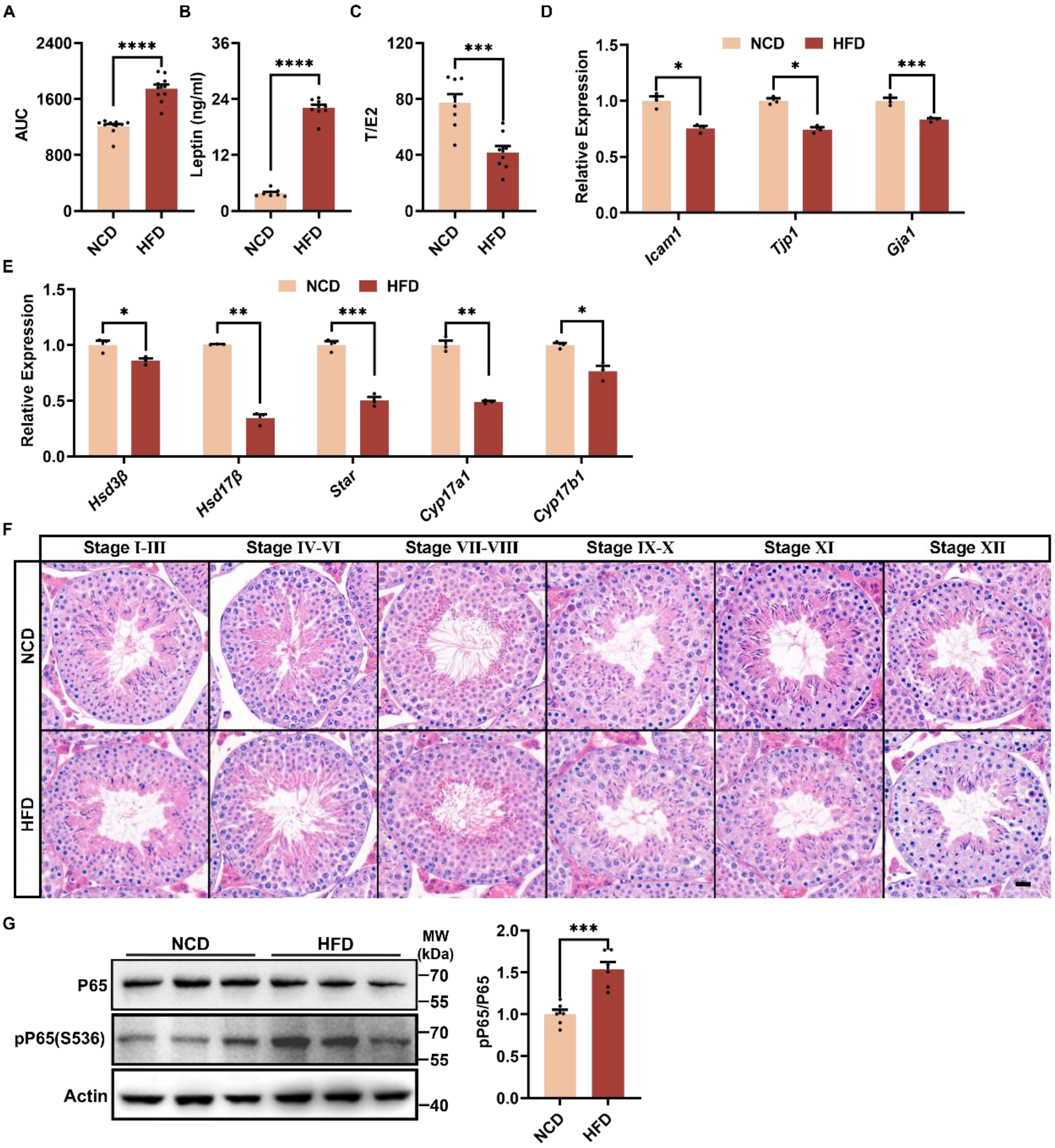

Supplemental Figure 2 Various metabolic and reproductive indexes were assessed in DIO mice

(A-C) Area under the glucose tolerance test (GTT) curves (A), serum leptin levels (B), T/E2 ratios (C) of normal-chow diet (NCD) & high-fat diet (HFD) mice (n=10).

(D and E) The mRNA levels of blood-testis barrier (BTB) related genes including intercellular adhesion molecule 1 (*Icam1*), tight junction protein 1 (*Tjp1*) and gap junction protein alpha 1 (*Gja1*) (D), and genes associated with the synthesis of testosterone in cluding hydroxy-delta-5-steroid dehydrogenase 3 beta (*Hsd3β*), hydroxy-delta-5-steroid dehydrogenase 17 beta (*Hsd17β*), steroidogenic acute regulatory protein (*Star*), cytochrome P450 family 11 subfamily A member 1 (*Cyp11a1*) and cytochrome P450 family 11 subfamily B member 1 (*Cyp11b1*) (E), of NCD & HFD mice (n=3).

(F) Representative Hematoxylin-eosin (H&E) staining images of testicular tissue from NCD & HFD mice, scale bar = 20 μm.

(G) Western blot analysis of P65 and pP65 in hypothalamus of NCD & HFD (n=5) group male mice, and the quantification of the pP65 protein levels.

Data are presented as Mean ± SEM, * indicates a significant difference (* means $P < 0.05$, ** means $P < 0.01$, *** means $P < 0.001$), student's t-test.

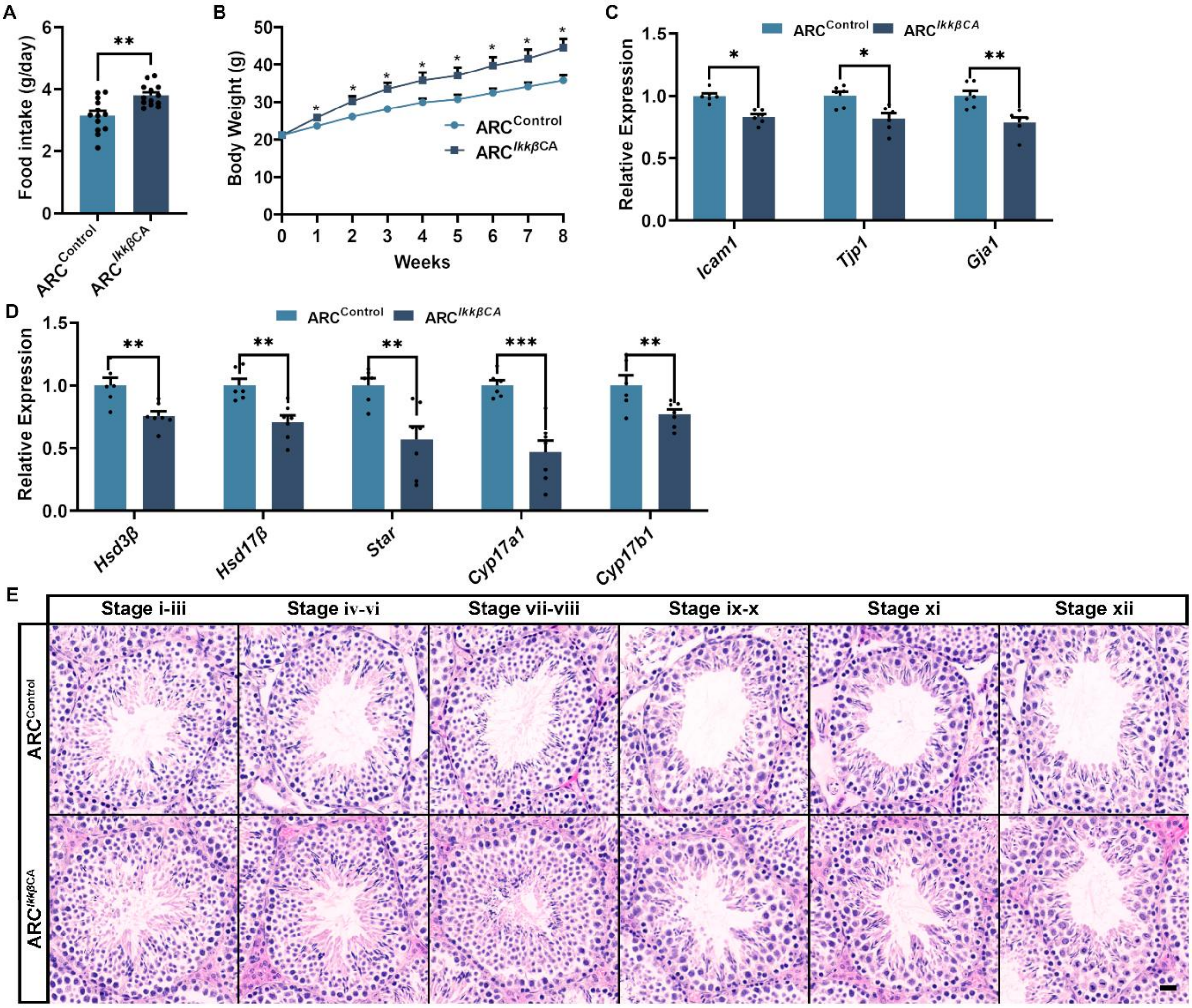

Supplemental Figure 3 Testicular dysfunction were assessed in ARC$^{Ikk\beta\mathrm{CA}}$ mice

(A and B) Food intake (n=13)(A) and Body weight (n=5)(B) in ARC^{Control} & ARC$^{Ikk\beta\mathrm{CA}}$ group .

(C and D)The mRNA levels of BTB related genes including *Icam1*, *Tjp1* and *Gja1* (C), and genes associated with the synthesis of testosterone including *Hsd3β*, *Hsd17β*, *Star, Cyp11a1* and *Cyp11b1* (D), in ARC^{Control} & ARC$^{Ikk\beta\mathrm{CA}}$ group (n=6).

(E) Representative H&E staining images of testicular tissue of ARC^{Control} & ARC$^{Ikk\beta\mathrm{CA}}$ mice, scale bar = 20 µm.

Data are presented as Mean ± SEM, * indicates a significant difference (* means $P < 0.05$, ** means $P < 0.01$, *** means $P < 0.001$), student's t-test.

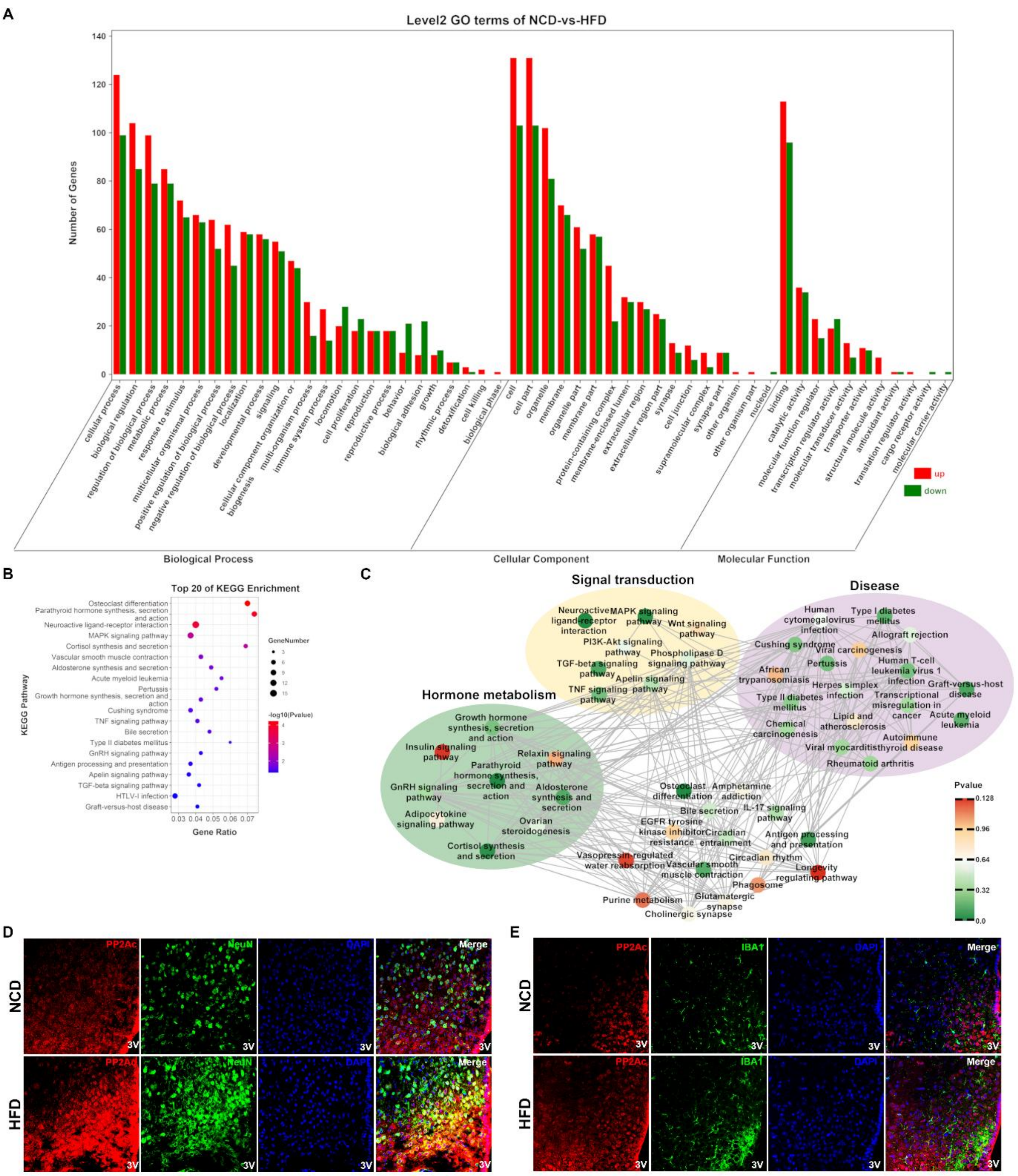

Supplemental Figure 4 Enrichment analysis of DEGs and localization of PP2Ac in the ARC

(A) Differentially expressed genes (DEGs) are enriched in various secondary terms.

(B) Top20 of Kyoto Encyclopedia of Genes and Genomes (KEGG) pathways.

(C) The majority of the top 50 signaling pathways in the network exhibit enrichment in Hormone metabolism, Signal transduction, and Disease.

(D) The representative fluroscence images shows the expression of PP2Ac (Red) in neurons (NeuN, Green) of arcuate nucleus (ARC) under NCD and HFD, scale bar= 20 μm.

(E) The representative fluroscence images shows the expression of PP2Ac (Red) in microglia (IBA1, Green) of ARC under NCD and HFD, scale bar= 20 μm.

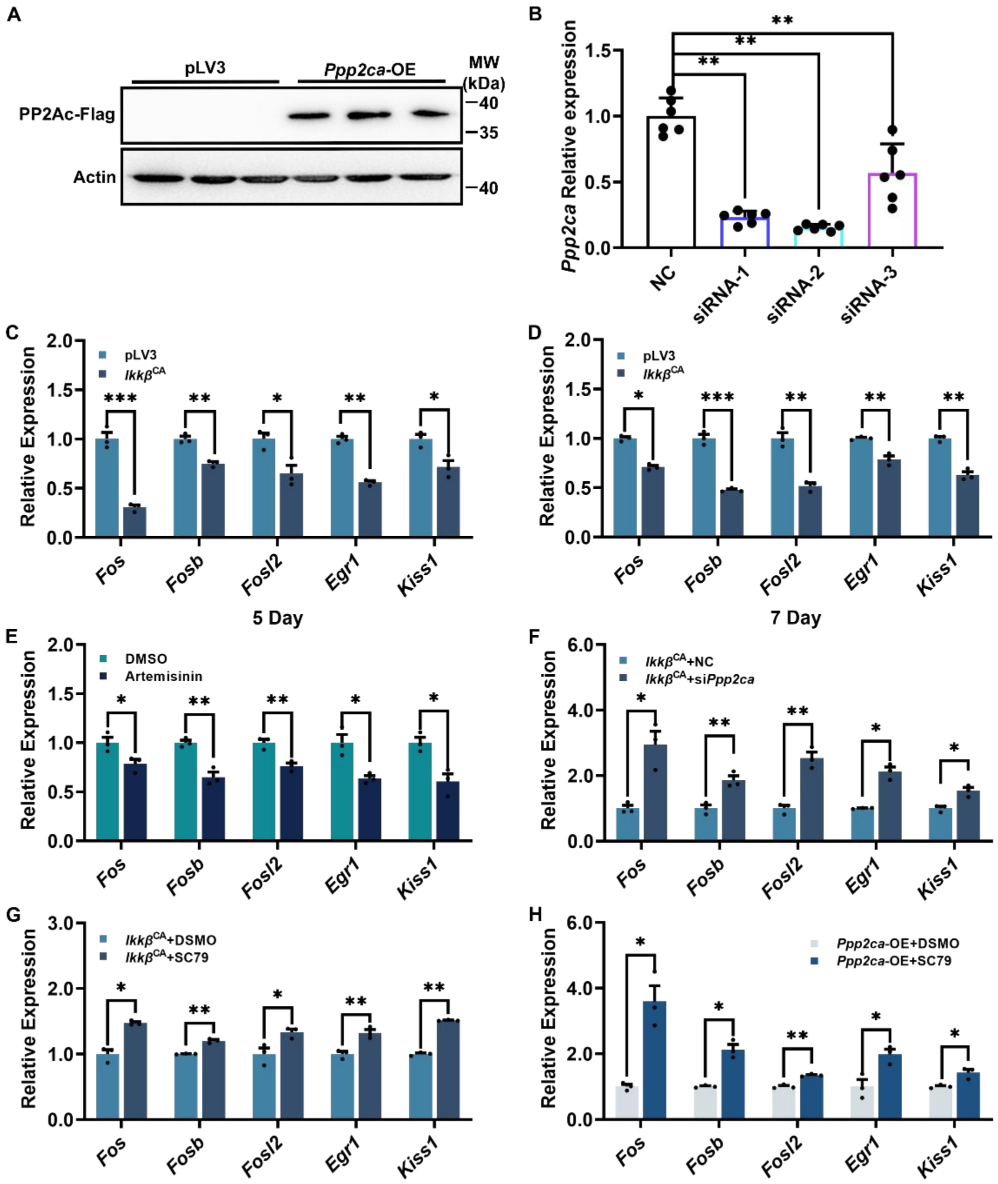

Supplemental Figure 5 Activation of NF-κB signaling induces upregulation of *Ppp2ca* expression and concomitantly reduction of phosphorylation levels of AKT and CREB1

(A) Western blot analysis of the expression of *Ppp2ca*-Flag in control cells and *Ppp2ca*-OE cells.

(B) Relative mRNA levels of *Ppp2ca* in N43/5 cells treated with NC, siRNA-1, siRNA-2 and siRNA-3 (n=6).

(C and D) Relative mRNA levels of FBJ osteosarcoma oncogene (*Fos*), FBJ osteosarcoma oncogene B (*Fosb*), FOS like 2, AP-1 transcription factor subunit (*Fosl2*), early growth response 1 (*Egr1*) and *Kiss1* in *Ikkβ*CA cells at Day 5 and Day 7 (n=3).

(E-H) Relative mRNA level of *Fos*, *Fosb*, *Fosl2*, *Egr1* and *Kiss1* in DMSO treated cells & Artemisinin treated cells (n=3) (E), in *Ikkβ*CA cells treated with NC & si*Ppp2ca* (F) (n=3) and DMSO & SC79 (G) (n=3), in *Ppp2ca*-OE cells treated with DMSO & SC79 (H) (n=3).

Data are presented as Mean ± SEM, * indicates a significant difference (* means $P < 0.05$, ** means $P < 0.01$, *** means $P < 0.001$), student's t-test.

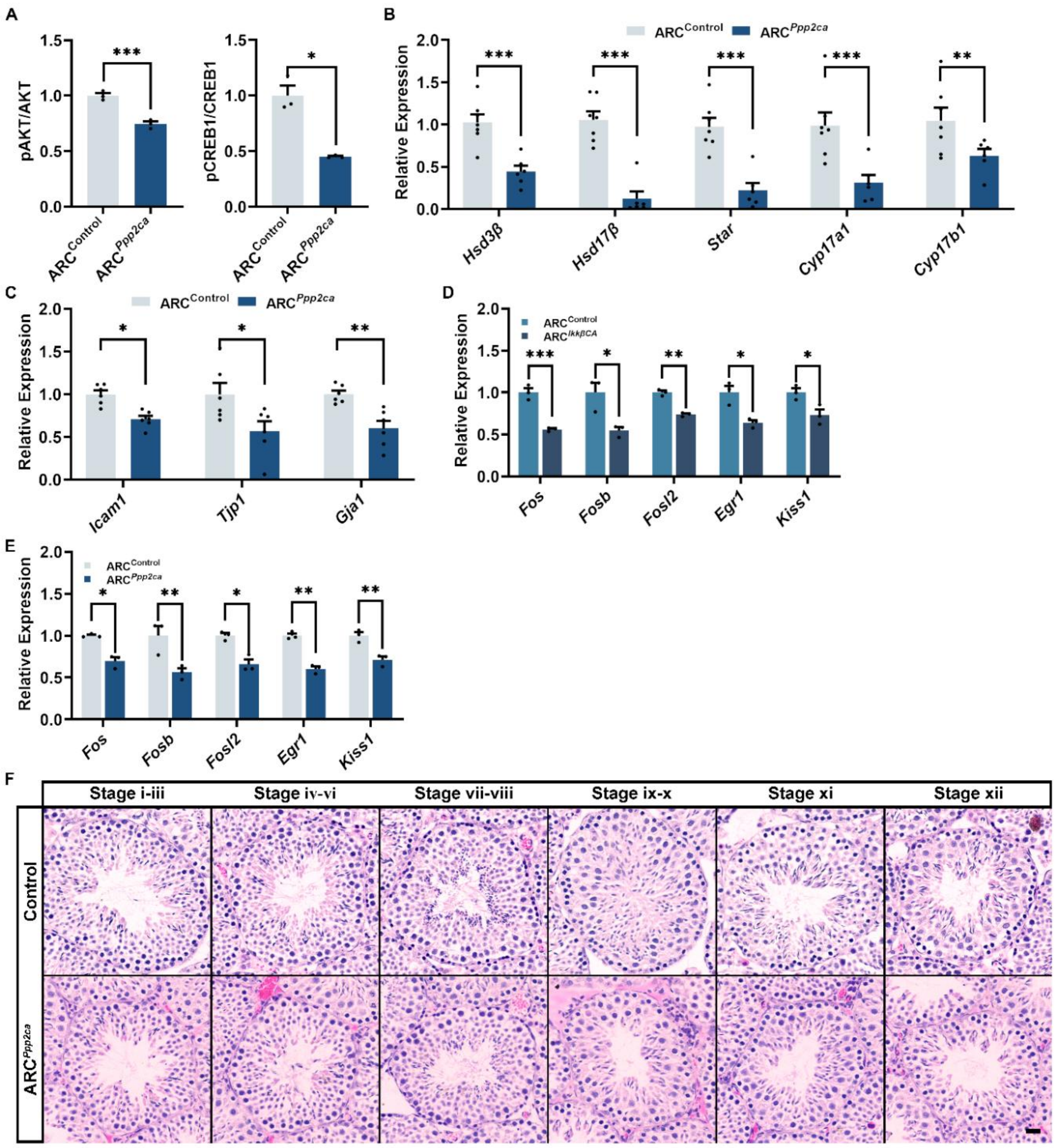

Supplemental Figure 6 Various reproductive indexes were assessed in ARC^{Ppp2ca} mice

(A) The quantification of pAKT and pCREB1 protein levels in hypothalamus of ARC^{Control} & ARC^{Ppp2ca} mice (n=3).

(B and C) The mRNA levels of the genes associated with testosterone synthesis, including *Hsd3β*, *Hsd17β*, *Star, Cyp11a1* and *Cyp11b1* (B) and BTB related genes including *Icam1*, *Tjp1* and *Gja1* (C) in the testis of ARC^{Control} & ARC^{Ppp2ca} mice (n=6).

(D and E) Relative mRNA levels of *Fos*, *Fosb, Fosl2*,, *Egr1* and *Kiss1* in the testis of ARC^{Control} & ARC^{IkkβCA} mice (n=3) (D), and in the testis of ARC^{Control} & ARC^{Ppp2ca} mice (n=3) (E).

(F) Representative H&E staining images of testicular tissue of ARC^{Control} & ARC^{Ppp2ca} mice, scale bar = 20 μm.

Data are presented as Mean ± SEM, * indicates a significant difference (* means $P < 0.05$, ** means $P < 0.01$, *** means $P < 0.001$), student's t-test.

Supplementary Table 4. Statistics of GSEA enrichment results

	Enrichment in NCD			Enrichment in HFD		
	**Upregulated**	**FDR< 0.25**	**Pvalue< 0.05**	**Upregulated**	**FDR< 0.25**	**Pvalue< 0.05**
H	27	1	2	23	6	5
C1	128	0	8	114	0	8
C2:cgp	1307	38	188	1352	58	158
C2:cp.Biocarta	83	0	4	60	0	1
C2:cp.reactome	219	1	23	271	16	26
C3:mir	164	1	31	36	0	0
C3:tft	401	70	76	170	0	5
C4:cgn	176	1	8	251	61	50
C4:cm	175	3	23	175	41	43
C6	104	16	20	84	0	5
C7	2084	122	245	2788	69	262

Note: The result of GSEA enrichment analysis by all genes have been identified. H:hallmark gene sets; C1: positional gene sets; C2: curated gene sets; cgp: chemical and genetic perturbations; cp: Canonical pathways; C3: regulatory target gene sets; mir: microRNA targets; tft: transcription factor targets; C4: computational gene sets; cgn: cancer gene neighborhoods; cm: cancer modules; C6: oncogenic signature gene sets; C7: immunologic signature gene sets.

Supplemental Table 5. List of Antibodies used in this study

Antibody	Company	Cat.NO	Source	Application	Dilution
LHβ capture antibody	University of California	518B7	Bovine	ELISA	1:5000
LH	Medix Biochemica	100588	Mouse	ELISA	1:500
poly-HRP conjugate streptavidin	Thermo Fisher	N200	/	ELISA	1:8000
Alexa Fluor Plus 555	Invitrogen	A32732	Goat	IF	1:500
Alexa Fluor Plus 488	Invitrogen	A32723	Goat	IF	1:500
PP2A-α	CST	2038	Rabbit	IF	1:2000
IBA1	abcam	ab283319	Mouse	IF	1:250
NeuN	abcam	ab104224	Mouse	IF	1:1000
HA	CST	3724	Rabbit	IF/WB	1:800/1:1600
Phospho-Akt (Ser473)	CST	4060	Rabbit	WB	1:1000
Akt	CST	4691	Rabbit	WB	1:1000
Phospho-NFκB p65 (Ser536)	Bioworlde	BS66162	Rabbit	WB	1:500
NF-κB p65	CST	8242	Rabbit	WB	1:1000
Phospho-CREB1 (Ser133)	Bioworlde	BS79369	Rabbit	WB	1:2000
CREB1	Proteintech	12208-1-AP	Rabbit	WB	1:2000
PP2A-α	Bioworlde	BS4867N	Rabbit	WB	1:2000
Flag	Bioworlde	AP007	Rabbit	WB	1:1000
β-Actin	Beyotime	AF0003	Mouse	WB	1:1000
α-Tubulin	Beytime	AF0001	Rabbit	WB	1:5000
HRP-labeled Goat Anti-Rabbit lgG(H+L)	Beyotime	A0208	Goat	WB	1:1000
HRP-labeled Goat Anti-Mouse IgG(H+L)	Beyotime	A0216	Goat	WB	1:1000

Supplemental Table 6. List of primers used in this study

Gene name	Primer sequence (5′- 3′)	Accession number
β-Actin	F: TATGCTCTCCCTCACGCCATCC	NM_007393.4
	R:GGAACCGCTCGTTGCCAATAGT	
Egr1	F: GCCAGGAGTGATGAACGCAAGA	NM_007913.4
	R:AGGAGCCAGGAGAGGAGTAGGA	
Fos	F: CAGCCGACTCCTTCTCCAGCAT	NM_010234.2
	R: TGCCGCCTGACATGGTCTTCA	
Fosl2	F: CCAGCAGAAGTTCCGGGTAG	NM_008037.3
	R: GTAGGGATGTGAGCGTGGATA	
Ppp2ca	F: ATGGACGAGAAGTTGTTCACC	NM_019411.3
	R: CAGTGACTGGACATCGAACCT	
Star	F: TCGCTTGGAGGTGGTGGTAGAC	NM_178260.3
	R: GGCCGTGTTCAGCTCTGATGAC	
Hsd17b	F: TCCTGAGCACTTCCGGTGAGAG	NM_008291.3
	R: TCATCGGCGGTCTTGGTCATCT	
Hsd3b	F: GGTGACAGGAGCAGGAGGGTTT	NM_008293.3
	R: TGACAGCAGCAGTGTGGATGAC	
Kiss1	F: GCTGCTGCTTCTCCTCTGTGTC	NM_178260.2
	R: CCAGGCTTGCTCTCTGCATACC	
Cyp11a1	F:GCTCAACCTGCCTCCAGACTTC	NM_019779.4
	R:CCTGCCAGCATCTCGGTAATGT	
Cyp11b1	F:CAGCCTGAAGTTCCTCCACACC	NM_001033229.3
	R:CCTCTGCCAGCTCTCGATACAC	
Gja1	F:CTGGTGGTGTCCTTGGTGTCTC	NM_010288.3
	R:GCTCGCTGGCTTGCTTGTTG	
Icam1	F:CCTGGAGACGCAGAGGACCTTA	NM_010493.3
	R:GCCGCTCAGAAGAACCACCTT	
Tjp1	F:AGCTGCCTCGAACCTCTACTCT	NM_001417372
	R:CCTGGTGGTGGAACTTGCTCAT	

Supplemental Tbale 7. List of siRNAs that designed targeting CDS of *Ppp2ca*

siRNA name	siRNA sequence (5'-3')
NC	Sense: CAACAAGAUGAAGAGCACCAA
	Anti-sense: UUGGUGCUCUUCAUCUUGUUG
siRNA-1	Sense: GCUUGUAGCUCUUAAGGUU
	Anti-sense: AACCUUAAGAGCUACAAGC
siRNA-2	Sense: GAACUUGACGACACUCUUA
	Anti-sense: UAAGAGUGUCGUCAAGUUC
siRNA-3	Sense: GGCAGAUCUUCUGUCUACA
	Anti-sense: UGUAGACAGAAGAUCUGCC
